# Systematic review of health state utility values in metastatic non-small cell lung cancer with a focus on previously treated patients

**DOI:** 10.1186/s12955-018-0994-8

**Published:** 2018-09-12

**Authors:** Noman Paracha, Ahmed Abdulla, Katherine S. MacGilchrist

**Affiliations:** 1 F. Hoffmann-La Roche AG, Basel, Switzerland; 2 Epidemica Ltd, Bicester, Oxfordshire, UK; 3 Present address: Digipharm, Zug, Switzerland

**Keywords:** Health state utility values (HSUVs), Health-related quality of life (HRQoL), Metastatic non-small cell lung cancer (mNSCLC), Multi-attribute utility instruments (MAUIs), Standard gamble (SG), Time trade-off (TTO), Systematic literature review

## Abstract

**Background:**

Health state utility values (HSUVs) are an important input to economic evaluations and the choice of HSUV can affect the estimate of relative cost-effectiveness between interventions. This systematic review identified utility scores for patients with metastatic non-small cell lung cancer (mNSCLC), as well as disutilities or utility decrements relevant to the experience of patients with mNSCLC, by treatment line and health state.

**Methods:**

The MEDLINE®, Embase and Cochrane Library databases were systematically searched (September 2016) for publications describing HSUVs in mNSCLC in any treatment line. The EQ-5D website, the School of Health and Related Research Health Utilities Database (ScHARRHUD) and major pharmacoeconomic and clinical conferences in 2015–2016 were also queried. Studies in adults with previously treated mNSCLC were selected for further analysis. The information extracted included study design, description of treatment and health state, respondent details, instrument and tariff, HSUV or (dis) utility decrement estimates, quality of study, and appropriateness for use in economic evaluations.

**Results:**

Of 1883 references identified, 36 publications of 34 studies were included: 19 reported EQ-5D scores; eight reported HSUVs from valuations of vignettes made by members of the public using standard gamble (SG) or time trade-off (TTO); two reported SG or TTO directly elicited from patients; two reported EQ-5D visual analogue scale scores only; one reported Assessment of Quality of Life instrument scores; one reported HSUVs for caregivers to patients with mNSCLC using the 12-item Short-Form Health Survey; and one estimated HSUVs based on expert opinion. The range of HSUVs identified for comparable health states showed how differences in study type, tariff, health state and the measures used can drive variation in HSUV estimates.

**Conclusions:**

This systematic review provides a set of published HSUVs that are relevant to the experience of adult patients previously treated for mNSCLC. Our review begins to address the challenge of identifying reliable estimates of utility values in mNSCLC that are suitable for use in economic evaluations, and also highlights how varying estimates result from differences in methodology.

**Electronic supplementary material:**

The online version of this article (10.1186/s12955-018-0994-8) contains supplementary material, which is available to authorized users.

## Background

Non-small cell lung cancer (NSCLC) is the most common form of lung cancer, occurring in 85–90% of lung cancer cases [1], and includes adenocarcinoma (40% of all lung cancers), squamous cell carcinoma (25–30%) and large cell carcinoma (10–15%) [2]. NSCLC is staged according to the American Joint Committee on Cancer/Union for International Cancer Control system [3], and measurement of lesions follows the Response Evaluation Criteria in Solid Tumors (RECIST) [4]. Approximately 40% of patients will have metastatic NSCLC (mNSCLC) at diagnosis [5], which includes cancers found in the lung and in the lymph nodes in the middle of the chest (defined as stage IIIA and IIIB; no distant metastasis), and cancers that have spread to both lungs or to another part of the body (defined as stage IV; distant metastasis) [6, 7].

Treatment is recommended according to the stage of mNSCLC, but treatment options are limited in the later stages of disease [7, 8]. Five-year survival rates are considerably lower in later than in earlier stages of NSCLC (stage IA, 45%; stage IIIA, 14%; stage IIIB, 5%; stage IV, 1%) [9]. Moreover, symptoms such as coughing and wheezing, chest pain, hoarseness and weight loss can severely reduce functional independence in patients with mNSCLC [10, 11]. Patient-reported health-related quality of life (HRQoL) provides an overall evaluation of health, well-being and daily functioning, and is impaired in patients with mNSCLC owing both to the disease and to treatment sequelae. Maintenance or improvement of HRQoL is an important treatment goal [12].

HRQoL can be expressed as a health state utility value (HSUV) ranging from 0 (death) to 1 (full health). If the health state is considered to be worse than death, health states can be valued at less than 0. Utility values are key drivers in cost-effectiveness analyses because estimates of quality-adjusted life-years (QALYs) are obtained by multiplying HSUVs for each health state by the time spent in that health state. Estimates of cost per QALY are highly sensitive to the choice of HSUV. It is therefore important to identify specifically those HSUVs that have been derived using methods acceptable to health technology assessment (HTA) authorities [13].

HSUVs can be derived using a range of instruments and techniques [14, 15]. In brief, instruments include: generic preference-based measures such as the EQ-5D-3 L [16] or EQ-5D-5 L [17], Health Utilities Index (HUI) [18], 6-dimension Short-Form Health Survey (SF-6D) [19], Assessment of Quality of Life instrument (AQoL) [20], 15-dimensional HRQoL measure [21], Quality of Well-Being scale [22], and multi-attribute utility instrument; as well as directly elicited standard gamble (SG), time trade-off (TTO) and visual analogue scale (VAS, e.g. EuroQoL VAS [EQ-VAS]). Mapping algorithms may also be used to convert values obtained from a condition-specific questionnaire to a generic preference-based measure; or to convert data from the 12- or 36-item Short-Form Health Survey (SF-12 or SF-36) to SF-6D [23]. Techniques may vary in terms of whose health is being measured (a patient’s or a caregiver’s), who responds to the questionnaire or, if using vignettes, who considers the health-state description (the patient regarding their own health, a patient with a different disease, the patient’s closest caregiver, another caregiver, a physician or another healthcare provider). For preference-based measures, variation can stem from who values the health state (e.g. UK general population sample) and which choice-based method is used in this valuation (SG or TTO).

HTA bodies including the UK National Institute for Health and Care Excellence (NICE) [24, 25], the Scottish Medicines Consortium (SMC) [26], the Canadian Agency for Drugs and Technologies in Health (CADTH) [27], the French Haute Autorité de Santé (HAS) [28] and the Australian Pharmaceutical Benefits Advisory Committee (PBAC) [29] have stated preferences for HSUV methodology. Across these agencies, there is a preference for HSUVs estimated using generic preference-based measures. NICE has a strong preference for EQ-5D, as this reduces variability induced when different instruments are used between different disease areas. Agencies also strongly prefer patients to be the respondents, as patients can best describe their own health state. Finally, valuation estimated using a country-specific general-population tariff via a choice-based elicitation technique such as SG or TTO is preferred, as this represents societal preferences.

This systematic review had three main aims: first, to identify HSUVs for adults with previously treated mNSCLC, by treatment line and health state, and to evaluate the relevance of each health state to patients, for example, line of treatment, adverse events (AEs), response status and prognostic factors; second, to identify relevant disutilities or utility decrements associated with adverse events (irrespective of line of treatment or health state). Finally, the suitability of the HSUVs according to HTA reference case was explored and the quality of the HSUVs assessed.

## Methods

### Study design and search strategy

A systematic review of HSUVs in mNSCLC was undertaken to identify HSUV studies in any treatment line. Studies, published either as full papers or as conference abstracts, in patients previously treated for mNSCLC were selected for further analysis. The following databases were searched: Embase (1974 to 7 September 2016); MEDLINE® (1966 to 7 September 2016); MEDLINE In-Process and e-publications ahead of print (database inception to 7 September 2016); and the Cochrane Library (including the Cochrane Database of Systematic Reviews, the Database of Abstracts of Reviews of Effects, the Cochrane Central Register of Controlled Trials, the National Health Service Economic Evaluation Database and the HTA database; 1968 to 7 September 2016).

**Fig. 1:**
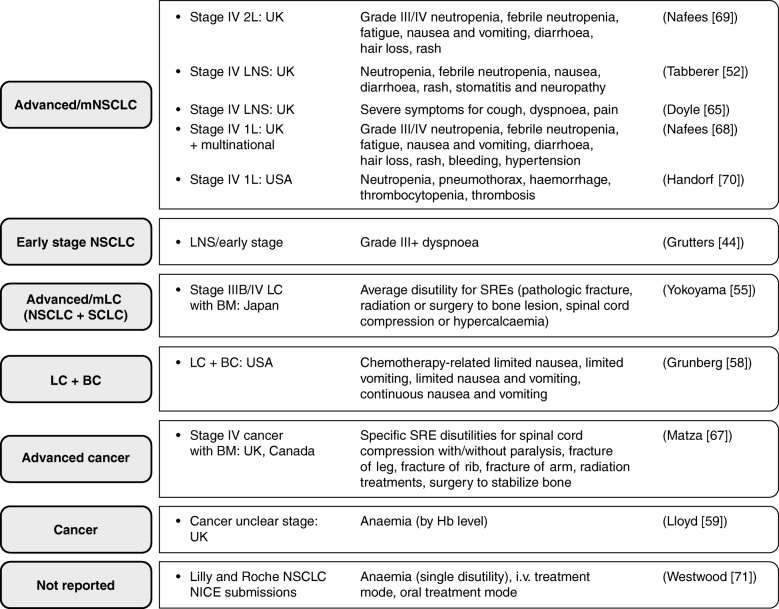
Studies reporting adverse event health state (dis) utilities by patient population and country. *Abbreviations: 1L* first line, *2L* second line, *BC* breast cancer, *BM* bone metastasis, *Hb* haemoglobin, *i.v.* intravenous, *LC* lung cancer, *LNS* line of treatment not specified, *mLC* metastatic lung cancer, *mNSCLC* metastatic non-small cell lung cancer, *NICE* National Institute for Health and Care Excellence, *NSCLC* non-small cell lung cancer, *SCLC* small cell lung cancer, *SRE* skeletal-related event

Search strings are summarized in Additional file 1: Table S1, and were constructed not only to find utilities in mNSCLC (using a wide range of NSCLC and mNSCLC terms combined with the HSUV filter adapted from Arber et al. 2015 [30]) but also to identify all relevant disutilities or utility decrements associated with AEs/comorbidities. To ensure that estimates would be available from previously treated mNSCLC populations for all AEs or comorbidity health states relevant to the experience of such patients, the strings were designed to search for disutilities or decrements from a broader group of populations, as follows: from lung cancer; for progressive disease disutilities from advanced/metastatic cancer; for disutilities associated with the most common sites of metastasis from the lung (bone, respiratory system, nervous system, adrenal gland and liver) from advanced cancer; for disutilities associated with AEs or toxicities of cancer therapy; and disutilities associated with specific grade 3–4 AEs known to occur with cancer treatments from advanced cancer populations (pneumonia, pneumonitis, increased aspartate aminotransferase, febrile neutropenia, neutropenia, infection, sepsis, fatigue, lethargy, nausea, vomiting, ulcers, stomatitis, gastrointestinal disturbance, diarrhoea, visual disturbance, hearing loss, hair loss, psychological/self-esteem changes, rash, anaemia, bleeding and hypertension). From the identified disutilities/decrements for each AE/co-morbidity health state, those from the most relevant population available could be selected following an order of decreasing population specificity from first-line mNSCLC to NSCLC, lung cancer and advanced/metastatic cancer (Fig. 1).

Using the term “NSCLC” or “non-small cell lung cancer”, manual searching of the EQ-5D website, of the School of Health and Related Research Health Utilities Database (ScHARRHUD) and of major pharmacoeconomic and clinical conferences in 2015–2016 was conducted on 3 and 5 December 2016. Conferences included: the International Society for Pharmacoeconomics and Outcomes Research (ISPOR) International Meetings and European Congresses; the HTA International Annual Meetings (HTAi); the Society for Medical Decision Making (SMDM) North American Meetings and European Conferences; the American Society of Clinical Oncology (ASCO) Meetings; and the European Society for Medical Oncology (ESMO) Congresses. Bibliographic reference lists of relevant systematic reviews from 2010 onwards were searched and of relevant cost-utility analyses, and HTA reports from various bodies identified in a parallel economic systematic review, including: NICE; SMC; All Wales Medicines Strategy Group (AWMSG); PBAC; CADTH; Institut National d’Excellence en Santé et en Services Sociaux; pan-Canadian Oncology Drug Review (pCODR); and HAS.

The PICOS (patient, intervention, comparator, outcome, study) statements for study inclusion and exclusion criteria are summarized in Table 1. Although, second- and later-line data were of primary interest, studies that reported utilities for patients with mNSCLC who were either treatment-naïve or in receipt of maintenance first-line treatment were included for reference at the first screening but data were not extracted. These studies are listed in Additional file 2: Table S2.

**Table 1:** Inclusion criteria

Characteristic	Inclusion criteria	Exclusion criteria
Population	Adult patients (aged ≥16 years) Locally advanced NSCLC or mNSCLC, second/subsequent line Locally advanced or metastatic NSCLC, line unspecified	Population not of interest, e.g. • in vitro data • animal data • mixed adult/child population or child population • mixed disease populations without mNSCLC data reported separately • not disease of interest • 1 L mNSCLC data (treatment-naïve or maintenance first-line treatment) were excluded but tagged^a^
Interventions/comparators	Not relevant for QoL SR selection. Intervention-specific utility data were noted as such during data extraction	N/A
Outcomes	For mNSCLC patients: • individual (patient or caregiver) derived mean or median health state utilities from indirect generic HRQoL measure (EQ-5D (-3 L and -5 L), SF-6D, HUI2, HUI3, AQoL, QWB, 15D, MAUI) or direct valuation by TTO, SG or EQ-VAS • SF-36 or SF-12 • general public valuations of vignettes using TTO or SG For NSCLC or wider population: • disutilities or decrements for AEs^b^ or progressive disease	No outcome of interest: • expert or healthcare provider (doctor, nurse) valuations of utilities • utilities not relating to a specific health state
Study design	RCTs, non-RCTs, observational data	Study design not of interest: • case reports, *n* = 1 before-and-after studies • PK/PD study only • (Non-systematic) reviews • SRs/NMAs^c^
Date limits	Unlimited	–
Child citation	Citation linked to another paper but with unique data	Child citation or sub-study with no unique data, determined at first or second pass
Duplicate citation		Duplicate/copy
Publication type		Publication type not of interest e.g. editorials, commentaries, letters, notes, press articles, unless relevant data has been published in a letter, for example, that does not appear elsewhere in the literature Confidential reports where unable to use report, or Hayes Inc. reports requiring purchase
Language	English or French Any foreign language paper with an English abstract were included if sufficient information is present in the English abstract to ensure the eligibility criteria are met	Full text in language other than English or French with no English abstract or no abstract; or insufficient information in English language abstract of foreign language full paper to assess eligibility

^a^To enable listing in the report

^b^Disutilities may be included for AEs, inconvenience of treatment or progressive health states from diseases outside NSCLC (preferably from lung cancer or from advanced/metastatic cancer) where no such data are available from patients with NSCLC

^c^SRs were kept in until the second pass, where the full paper’s included studies were examined, after which the SR itself was excluded

*Abbreviations*: *1 L* first line, *3 L* 3-level, *5 L* 5-level, *15D* 15-dimensional health-related quality of life measure, *AE* adverse event, *AQoL* Assessment of Quality of Life instrument, *EQ-VAS* EuroQoL visual analogue scale, *HRQoL* health-related quality of life, *HUI2/3* Health Utilities Index Mark 2/3, *MAUI* multi-attribute utility instrument, *mNSCLC* metastatic non-small cell lung cancer, *N/A* not available, *NMA* network meta-analysis, *NSCLC* non-small cell lung cancer, *PD* pharmacodynamic, *PK* pharmacokinetic, *QoL* quality of life, *QWB* Quality of Well-Being scale, *RCT* randomized controlled trial, *SF-6D* 6-dimension Short-Form Health Survey, *SF-12/36* 12/36-item Short-Form Health Survey, *SG* standard gamble, *SR* systematic review, *TTO* time trade-off*, VAS* visual analogue scale

Mapping from condition-specific to preference-based studies was not sought because it was anticipated that sufficient published utility and EQ-5D data would be available to populate the health states of an economic model, and because results based on mapping algorithms sit lower in the acceptance hierarchy used by some HTA authorities (Additional file 3: Figure S1). We have acknowledged NICE’s stated preference for EQ-5D-3 L data over EQ-5D-5 L (Additional file 3: Figure S1) and provide detailed information of the instrument used for generating data for each identified study in Table 2 [31].

**Table 2:** Identified utility studies by line of treatment

Author, year, country	Line of treatment	Health state	Instrument	Treatment
First line^a^				
Handorf 2012 USA [70]	1 L	Stage IV adenocarcinoma SDis, PD, SDis+AEs (neutropenia, pneumothorax, haemorrhage, thrombocytopenia, thrombosis)	Expert opinion estimates,^b^ published sources	NTS
Nafees 2016 Multinational and UK [68]	1 L	Metastatic NSCLC common grade III/IV toxicities (neutropenia, febrile neutropenia, fatigue, nausea and vomiting, diarrhea, hair loss, rash), bleeding, hypertension	TTO (general public)	NTS
≥ First line^c^				
Chevalier 2013 France and nine other countries [38]	1 L, 2 L, 3/4 L and BSC	Advanced/metastatic NSCLC 1 L, 2 L, 3/4 L PF and PD	EQ-5D	NTS
Chouaid 2013 Multinational [39]	1 L 55.1% 2 L 24.7% 3/4 L 17.9% BSC 2.3%	Advanced/metastatic NSCLC 1 L, 2 L, 3/4 L, BSC and mixed line PF and PD	EQ-5D, EQ-VAS	NTS
Iyer 2013 France, Germany [46]	1 L 52% 2 LL 48%	Advanced/metastatic NSCLC	EQ-5D	NTS
Second line				
Blackhall 2014 Multinational [41]	2 L after progression on platinum-based 1 L therapy	Locally advanced/metastatic ALK+ NSCLC BL and treatment-specific utilities (not PSS)	EQ-5D EQ-VAS	CRZ PEM DOC
Huang 2016 Worldwide [45]	2 L after platinum-based therapy	Advanced PD-L1+ NSCLC NTS PF, PD NTS time to death	EQ-5D	PEMB DOC
Langley 2013 UK, Australia [48]	2 L^d^	Treatment-specific stage IV NSCLC with BM at BL and after certain time points on treatment	EQ-5D	OSC WBRT + OSC
Nafees 2008 UK [69]	2 L	Metastatic NSCLC PD, RES, SDis, common grade III/IV toxicities (neutropenia, febrile neutropenia, fatigue, nausea and vomiting, diarrhea, hair loss, rash)	SG (general public)	NTS
Novello 2015 Multinational [49]	2 L	Stage III/IV recurrent NSCLC (SQ and NSQ) treatment-specific at BL and certain time points on treatment (≤ 30 weeks)	EQ-5D, EQ-VAS	NIN + DOC PLA + DOC
Reck 2015 Multinational [50]	2 L	Advanced SQ NSCLC treatment-specific at BL reported. Collected also for up to 1 year but values NR in abstract	EQ-5D, EQ-VAS	NIVO DOC
Rudell 2016 USA, Canada, Hong Kong, Italy, Japan, Republic of Korea, Spain, Taiwan [57]	2 L	Advanced EGFR+ NSCLC, treatment-specific at BL and 36 weeks on OSI	EQ-VAS	OSI
Schuette 2012 Germany, Austria [51]	2 L	Stage III/IV NSCLC treatment-specific at BL, 6 weeks (second cycle) and sixth cycle	EQ-5D EQ-VAS	PEM
Vargas 2009 Mexico [72]	2 L after previous CHEMO	NSCLC, stage NR (assumed advanced), treatment-specific not PSS	Global QoL index	ERL Taxanes
≥ Second line				
Chen 2010 UK/multinational [64]	2 L, 3 L and BSC	Stage IIIb/IV EGFR+ NSCLC treatment-specific (not PSS) On/after DOC 2 L On/after PEM 2 L On ERL 3 L BSC	SG (general public)	DOC PEM ERL BSC
Griebsch 2014 Multinational [37]	2LL^e^ and treatment-naïve	Stage IIIb with pleural effusion or stage IV NSCLC adenocarcinoma treatment-specific and NTS effect of progression	EQ-5D, EQ-VAS	AFA BSC CIS/PEM
Hirsh 2013 Multinational [40]	2LL^f^	Stage IIIb/IV NSCLC BL and treatment-specific on oral AFA 50 mg q.d. + BSC or PLA + BSC	EQ-5D	AFA + BSC PLA + BSC
Stewart 2015 Canada [56]	Targeted therapy 84% 3LL 25% RCT 22%	Metastatic EGFR+ NSCLC, all patients not PSS PR/SDis EGFR TKI RES CHEMO RES GEF RES ERL RES OSI PD EGFR TKI	EQ-5D-3 L	GEF ERL OSI
Schwartzberg 2015 USA, Canada [60]	2 LL	Squamous and non-squamous stage IIIb/IV NSCLC treatment-specific weeks 6–30 Treatment-specific PR, SDis and PD weeks 6–30	EQ-VAS	NIVO
Treatment line not specified				
Bradbury 2008 Canada [42]	Unclear	Advanced NSCLC Treatment-specific (not PSS)	EQ-5D	ERL BSC
Chang 2016 South Korea [63]	NR	Advanced NSCLC from > 360 days before death to < 30 days before death (not PSS)	TTO (general public)	NTS
Dansk 2016 UK [43]	NR	Synthesized advanced NSCLC PF, PD used in NICE HTAs Trial-based PF, PD Non-trial based PF, PD	EQ-5D	NTS
Doyle 2008 UK [65]	NR	Metastatic NSCLC SDis, RES, severe symptoms (cough, dyspnoea, pain)	SG (general public)	NTS
Grunberg 2009 USA [58]	NR	Mixed cancer population chemotherapy-related nausea, vomiting, and nausea and vomiting, of different severities	SG (patient)	CHEMO
Grutters 2010 Netherlands [44]	NR	NSCLC with grade 3+ dyspnoea	EQ-5D	NTS
Jang 2010 Canada [47]	NR	Stage IV NSCLC and locally advanced NSCLC	EQ-5D	NTS
Linnet 2015 Denmark [62]	Unclear	Metastatic NSCLC second and third CHEMO cycles on oral VINO Patient and caregiver utilities reported	SF-12	VINO
Lloyd 2005 UK [66]	NR	Stage IV NSCLC RES, SDis i.v. treatment, SDis oral treatment, PD, end of life	SG (general public)	NTS
Lloyd 2008 [59]	Previous CHEMO	Anaemia by haemoglobin level	General public SG, patient TTO	NTS
Manser 2006 Australia [61]	NR	Stage IV NSCLC	AQoL	NTS
Matza 2014 UK and Canada [67]	NR	Stage IV cancer with BMs and different types of SRE (spinal cord compression with/without paralysis, fracture of leg, fracture of rib, fracture of arm), radiation treatment (2 weeks, 5 appointments/week), radiation treatment (2 appointments), surgery to stabilize bone	TTO (general public)	NTS
Tabberer 2006 UK [52]	NR	Advanced NSCLC RES, SDis, SDis oral treatment, SDis i.v. treatment, PD, near death, AEs (neutropenia, febrile neutropenia, nausea, diarrhoea, rash, stomatitis, neuropathy)	EQ-5D (general public)	NTS
Trippoli 2001 Italy [53]	NR	Metastatic NSCLC	EQ-5D, EQ-VAS	NTS
Westwood 2014 [71]	NR for other disutilities	Advanced NSCLC Disutility for anaemia and for i.v./oral treatment mode	SG NR for other disutilities	NTS ERL i.v. tx
Yang 2014 Taiwan [54]	NR	NSCLC operable (I–IIIA) and NSCLC inoperable (IIIB/IV)	EQ-5D	NTS
Yokoyama 2013 Japan [55]	NR	Stage IIIB/IV mixed NSCLC/SCLC with bone metastasis and SRE (pathologic fracture, radiation or surgery to bone lesion, spinal cord compression or hypercalcaemia)	EQ-5D	NTS

^a^Studies were retained, despite reporting first-line treatment only, because they reported progressive disease utility estimates similar to those seen in a second-line population, or reported AE disutility estimates from populations broader than mNSCLC

^b^Although the utilities were based on expert opinion, these were retained, as they provide disutility estimates for the adverse events pneumothorax, thrombocytopenia and thrombosis, not available elsewhere

^c^Studies reported data on first-line treatment and subsequent treatment lines

^d^Previous treatment with systemic CHEMO or EGFR inhibitors allowed

^e^Lux-Lung 1 trial data were in patients progressed on 1–2 lines of treatment, one of which was platinum based (could include adjuvant setting treatment line), and had PD after at least 12 wks of ERL or GEF. Lux-Lung 3 trial data were in treatment-naïve patients, so not 2 L.

^f^Progressed on 1–2 lines of treatment, one of which was platinum based, and had PD after at least 12 wks of ERL or GEF

*Abbreviations: 1 L* first line, *2 L* second line, *2 LL* second and subsequent line, *3LL* third and subsequent line, *3/4 L* third and fourth line, *AE* adverse event, *AFA* afatinib, *AQoL* Assessment of Quality of Life instrument, *BL* baseline, *BM* bone metastasis, *BSC* best supportive care, *CHEMO* chemotherapy, *CIS* cisplatin, *CRZ* crizotinib, *DOC* docetaxel, *EGFR* epidermal growth factor receptor, *EQ-VAS* EuroQol visual analogue scale, *ERL* erlotinib, *GEF* gefitinib, *GEM* gemcitabine, *i.v.* intravenous, *NIN* nintedanib, *NIVO* nivolumab, *NR* not reported, *NSCLC* non-small cell lung cancer, *NSQ* non-squamous, *NTS* not treatment-specific, *OSC* optimal standard care, *OSI* osimertinib, *PD* progressive disease, *PEM* pemetrexed, *PEMB* pembrolizumab, *PF* progression-free, *PLA* placebo, *PR* partial response, *PSS* progression-status-specific, *q.d.* once daily, *QoL* quality of life, *RCT* randomized controlled trial, *RES* response, *SCLC* small cell lung cancer, *SDis* stable disease, *SF-12* 12-item Short–Form Health Survey, *SG* standard gamble, *SRE* skeletal-related event, *TKI* tyrosine kinase inhibitor, *TTO* time trade-off, *VAS* visual analogue scale, *VINO* vinorelbine, *WBRT* whole-brain radiotherapy

### Study selection

The screening process complied with the 2009 Preferred Reporting Items for Systematic Reviews and Meta-Analyses (PRISMA) guidelines [32]. Publications were de-duplicated using EndNote (Clarivate Analytics, Philadelphia, PA, USA) and using Rayyan (Qatar Computing Research Institute, Doha, Qatar) [33], an internet-based reference management system endorsed as suitable for systematic review screening by the European Network for HTA [34]. Abstracts and titles of papers were screened by one reviewer, and a 50% sample check conducted by a second reviewer; exclusion criteria are summarized in Table 1. The full texts of papers potentially meeting the selection criteria were screened by one reviewer, and a 50% sample check was conducted by a second reviewer. Discrepancies were discussed between reviewers, and any unresolved disputes were referred to a third reviewer.

### Data extraction

Data were collected using a piloted data-extraction sheet. Extraction was conducted by one reviewer, and priority data elements were quality checked by a second reviewer. The information extracted included study design, whether the selection criteria yielded a population that matched the target population (i.e. previously treated adult patients with mNSCLC), health state description, instrument type, instrument scale, HSUV or (dis) utility or decrement estimates and measure of variability (median with interquartile range or mean with standard error, standard deviation or 95% confidence interval), derivation methods and if the data presented were appropriate for use in HTA submissions to NICE, SMC, CADTH, HAS and PBAC.

### Quality and relevance assessment

The appropriateness of utilities reported for use in economic evaluations was determined by whether data met the requirements of the HTA body reference case; and the quality of utility estimates (based on sample size, response to the questionnaire, loss to follow-up, handling of missing data, and reporting of point and variance estimates, as discussed in NICE Decision Support Unit Technical Support Document 11 and its related publication [25, 35]; Additional file 4: Table S3). Any recommendation for, or rationale against, the use of specific utilities in a cost–utility analysis model in previously treated patients with mNSCLC was also taken into consideration in line with preliminary guidance from the ISPOR Health State Utility Good Practices Task Force [36].

## Results

### Search yields

**Fig. 2:**
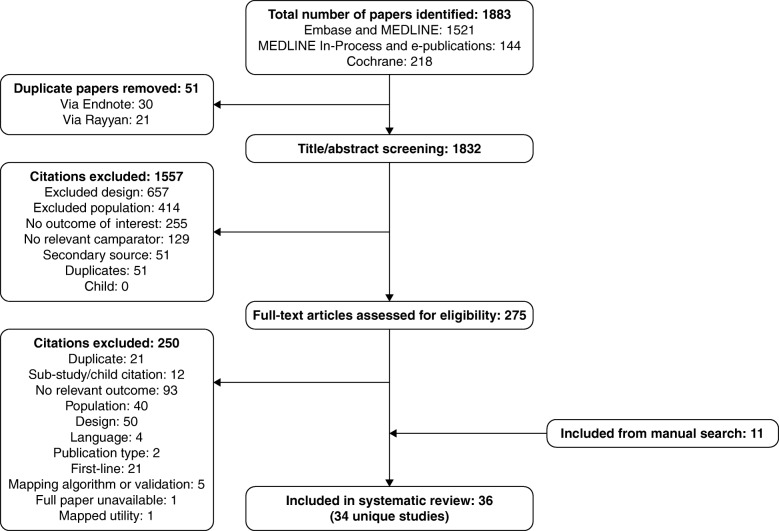
PRISMA flow chart for study selection. *Abbreviation: PRISMA* Preferred Reporting Items for Systematic Reviews and Meta-Analyses

Electronic database searches identified 1883 citations (1521 from MEDLINE/Embase, 144 from MEDLINE In-Process/e-publications and 218 from the Cochrane Library databases). After de-duplication (51 citations: 30 via Endnote and 21 via Rayyan) and title/abstract screening (1557 exclusions), 275 full-text papers were reviewed. Of these, 250 were excluded (21 of which were tagged as reporting first-line treatment; Additional file 2: Table S2), yielding 25 citations that were included from electronic sources. Manual searching identified 11 citations. In total, 36 articles were included, reporting 34 studies (Table 2). Two articles [37, 38] were linked to other publications [39, 40], and were retained because they provided additional information. The study selection is summarized in a PRISMA flow chart in Fig. 2.

### Description of studies identified

Among the 36 articles (34 studies) identified, 19 reported EQ-5D scores [37–55] (three studies further specified the instrument as EQ-5D-3 L [39, 41, 56] and two as EQ-5D-5 L [44, 57]; Table 2), two reported SG or TTO directly elicited from patients [58, 59], two reported EQ-VAS scores only [57, 60], and one reported AQoL scores [61] (Table 2). Moreover, one study reported SF-12 scores for caregivers to patients with mNSCLC [62], eight reported HSUVs from valuations of vignettes made by members of the public using SG or TTO [59, 63–69] (one of which reported both general public-elicited SG and patient-elicited TTO) [59], and one reported disutility estimates based on expert opinion for pneumothorax, thrombocytopenia and thrombosis, adverse event health states for which disutilities were not available from other HSUV derivation methods [70]. A further two articles reported HSUVs but were unclear about how these were derived; one reported disutilities used in previous NICE submissions, for anaemia and for oral and intravenous treatment modes [71], and one reported a “global quality of life index” for second-line NSCLC [72].

Among the dataset, two studies were retained despite reporting first-line treatment only, because they reported AE disutility estimates from populations broader than mNSCLC [68, 70]; three further studies that reported first-line data also reported on subsequent treatment lines [38, 39, 46]. Eleven studies focused exclusively on HSUVs associated with second-line treatment [41, 45, 48–51, 57, 59, 69, 71, 72], and five reported HSUVs in second-line and subsequent treatment [37, 40, 56, 60, 73]. Line of treatment was unspecified in 15 studies [42–44, 47, 52–55, 58, 61–63, 65–67].

### Relevant HSUVs by line of treatment

Utilities were reported for a range of health state types: treatment-specific or not, RECIST response-based or not, time-on-treatment, time-till-death, or a combination of these. Details of HSUV estimates by treatment line are given in Table 3. Among patients receiving second-line or subsequent treatment for advanced NSCLC or mNSCLC, mean HSUV estimates based on EQ-5D for stable/progression-free disease and for patients at baseline or pre-treatment were in the range 0.66–0.76 [38, 39, 41, 45, 49, 50]; in the same group, mean values for patients with progressive disease were generally lower (0.55–0.69) [38, 39, 45]. Among patients on treatment at this stage of disease and treatment line, the range of mean HSUVs based on EQ-5D was broad (0.53–0.82) [40, 41, 46, 51, 56], the highest value being associated with treatment with tyrosine kinase inhibitors [41, 56]. A similar range of HSUV values was seen among patients being treated for advanced NSCLC or mNSCLC when the treatment line was unspecified (0.53–0.77) [42, 47, 52, 53]. Only three papers specified using EQ-5D-3 L [39, 41, 56] and only two EQ-5D-5 L [44, 57].

**Table 3:** Health state utility values by treatment line, health state and instrument

Study	Health state	Utility value^a^	Instrument	Tariff	Respondent details	HTA suitability
1st line						
Nafees 2016 [68] Metastatic NSCLC^b^	PD vs BL state	0.095	TTO	N/A	Patients (but not NSCLC patients) from the general public in UK, Australia, France, China, S. Korea, Taiwan	No
	RES no side effects vs BL	0.773				
	SDis no side effects vs BL	0.460				
Chevalier 2013^c^ [38] Advanced/metastatic NSCLC	1 L PF	0.69 (0.26)	EQ-5D	French (TTO)	Stage I–Stage III/IV PF and PD	Meets HAS requirements
	1 L PD	0.61 (0.24)				
Chouaid 2013 [39] Advanced/metastatic NSCLC	1 L PF	0.71 (0.24) (95% CI, 0.67–0.76)	EQ-5D-3 L	UK	At time of advanced diagnosis, mean age 64.8 years Adenocarcinoma: 65.2% Large-cell carcinoma: 6.8% SQ cell carcinoma: 17.1% Other: 9.9% Clinical stage at time of survey: IIIb: 17.9% IV: 82.1%	Meets NICE requirements
		69.31 (18.33) (95% CI, 65.9–72.8)	EQ-5D VAS	N/A		No
	1 L PD	0.67 (0.2) (95% CI 0.59–0.75)	EQ-5D-3 L	UK		Meets NICE requirements
		58.67 (17.4) (95% CI 51.3–66.0)	EQ-5D VAS	N/A		No
Iyer 2013 [46] Advanced/metastatic NSCLC	France, Germany 1 L	0.63 (0.31)	EQ-5D	UK1	Patients with: Adenocarcinoma: 56.3% Large-cell carcinoma: 11.8% SQ cell carcinoma: 29.3% Other: 2.5% Stage IIIb: 15.4% Stage: IV 84.6%	Meets NICE requirement
		60.8 (19.9)	EQ-5D VAS	N/A		No
≥ 1st line						
Iyer 2013 [46] Advanced/metastatic NSCLC	France, Germany 1 L/2 L	0.58 (0.35)	EQ-5D	UK	Patients with: Adenocarcinoma: 56.3% Large-cell carcinoma: 11.8% SQ cell carcinoma: 29.3% Other: 2.5% Stage IIIb: 15.4% Stage: IV 84.6%	Meets NICE and SMC requirements
	France, 1 L/2 L	0.57 (0.41)				
	Germany, 1 L/2 L	0.59 (0.31)				
	France, Germany 1 L/2 L	58.0 (19.9)	EQ-5D VAS			No
	France, 1 L/2 L	57.1 (21.1)				
	Germany, 1 L/2 L	58.6 (19.1)				
2nd line						
Blackhall 2014 [41] Advanced/metastatic ALK+ NSCLC	2 L BL CRZ	0.73 (0.24)	EQ-5D-3 L	NR	Multinational patients, locally advanced/metastatic ALK+ NSCLC, 2 L	Unclear as tariff NR
	2 L BL chemotherapy (PEM or DOC)	0.70 (0.26)				
	2 L BL PEM	0.73 (0.24)				
	2 L BL DOC	0.67 (0.29)				
	2 L on CRZ	0.82 (SE, 0.01) (95% CI, 0.79–0.85)				
	2 L on Chemotherapy	0.73 (SE, 0.02) (95% CI, 0.70–0.77)				
	2 L on PEM	0.74 (SE, 0.02) (95% CI, 0.70–0.79)				
	2 L on DOC	0.66 (SE, 0.04) (95% CI, 0.58–0.74)				
Chevalier 2013^c^ [38] Advanced/metastatic NSCLC	2 L PF	0.70 (0.22)	EQ-5D	French (TTO)	Stage I–Stage III/IV PF and PD	Meets HAS requirements
	2 L PD	0.55 (0.35)				
Chouaid 2013 [39] Advanced/metastatic NSCLC	2 L PF	0.74 (0.18) (95% CI, 0.68–0.80)	EQ-5D-3 L	UK	At time of advanced diagnosis, mean age 64.8 years Adenocarcinoma: 65.2% Large-cell carcinoma: 6.8% SQ cell carcinoma: 17.1% Other: 9.9% Clinical stage at time of survey: IIIb: 17.9% IV: 82.1%	Meets NICE requirements
		65.0 (19.6) (95% CI, 59.2–70.8)	EQ-5D VAS	N/A		No
	2 L PD	0.59 (0.34) (95% CI, 0.42–0.77)	EQ-5D-3 L	UK		Meets NICE requirements
		53.5 (23.3) (95% CI, 41.5–65.4)	EQ-5D VAS	N/A		No
Huang 2016^c^ [45] Advanced PD-L1+ NSCLC	2 L PF	0.76 (95% CI, 0.75–0.77)	EQ-5D	NR	Multinational patients with advanced NSCLC and PD-L1+ tumours in 2 L on PEMB or DOC, after platinum-based chemotherapy	Unclear as tariff NR
	2 L PD	0.69 (95% CI, 0.66–0.71)				
	Advanced PD-L1+ NSCLC, 2 L, > 360 days from death	0.81 (0.79, 0.83)			Patients with advanced NSCLC and PD-L1+ tumours in 2 L on PEMB or DOC, after platinum-based chemotherapy	
	Advanced PD-L1+ NSCLC, 2 L, 180–360 days from death	0.73 (0.71, 0.75)				
	Advanced PD-L1+ NSCLC, 2 L, 90–180 days from death	0.69 (0.66, 0.72)				
	Advanced PD-L1+ NSCLC, 2 L, 30–90 days from death	0.60 (0.56, 0.64)				
	Advanced PD-L1+ NSCLC, 2 L, < 30 days from death	0.40 (0.31, 0.48)				
Iyer 2013 [46] Advanced/metastatic NSCLC	On treatment: 2 L only	0.53 (0.38)	EQ-5D	UK	French and German patients	Meets NICE and SMC requirements
		54.9 (19.3)	EQ-5D VAS	N/A		No
Langley 2013 [48] Stage IV NSCLC with brain metastases	NSCLC with BM, previous tx allowed, OSC + WBRT 0 days	0.63	EQ-5D	NR^d^	UK and Australian NSCLC patients with brain metastases	No, as VAS tariff used
	NSCLC with BM, previous tx allowed, OSC + WBRT 28 days	0.49				
	NSCLC with BM, previous tx allowed, OSC + WBRT 56 days	0.39				
	NSCLC with BM, previous tx allowed, OSC + WBRT 112 days	0.36				
	NSCLC with BM, previous tx allowed, OSC + WBRT 168 days	0.16				
	NSCLC with BM, previous tx allowed, OSC alone 0 days	0.60				
	NSCLC with BM, previous tx allowed, OSC alone 28 days	0.49				
	NSCLC with BM, previous tx allowed, OSC alone 56 days	0.44				
	NSCLC with BM, previous tx allowed, OSC alone 112 days	0.38				
	NSCLC with BM, previous tx allowed, OSC alone 168 days	0.36				
Lloyd 2008 [59] Cancer with chemotherapy-related anaemia or fatigue	Anaemia, Hb level, ≥12.0 g/dL	0.708 (95% CI, 0.057)	SG	N/A	General public sample from UK	No
		0.611 (95% CI, 0.112)	TTO		UK cancer patients who have recently experienced chemotherapy-related fatigue and anaemia completing vignette-based TTO	Meets NICE/SMC requirements but still vignette-based health state rather than patient rating own health
Nafees 2008 [59] mNSCLC	2 L Stable disease^e^	0.65 (SE, 0.02)	SG	N/A	100 members of general public in UK	No, but used in multiple HTA submissions
	2 L Responding disease^f^	0.67				
	2 L Response gain	0.02 (SE, 0.01)				
	2 L Progressive disease^g^	0.47				
Novello 2015 [49] Stage III/IV recurrent NSCLC (SQ and NSQ)^h^	2 L NIN + DOC, before treatment (week 0)	0.72	EQ-5D	UK	Multinational patients with stage III/IV recurrent NSCLC (SQ and NSQ) in 2 L after chemotherapy Adenocarcinoma: 50.1%	Meets NICE/SMC requirements
	2 L NIN + DOC, after treatment (week 30)	0.61				
	2 L PLA + DOC, before treatment (week 0)	0.72				
	2 L PLA + DOC, after treatment (week 30)	0.62				
	2 L NIN + DOC, before treatment (week 0)	69.0	EQ-5D VAS	N/A		No
	2 L NIN + DOC, after treatment (week 30)	63.2				
	2 L PLA + DOC, before treatment (week 0)	69.0				
	2 L PLA + DOC, after treatment (week 30)	63.1				
Reck 2015 [50] Advanced SQ NSCLC	2 L NIVO at BL	0.68 (0.208)	EQ-5D	NR	Multinational patients with advanced SQ NSCLC	Unclear as tariff NR
	2 L DOC at BL	0.66 (0.284)				
	2 L NIVO at BL	63.7 (18.2)	EQ-5D VAS	N/A		No
	2 L DOC at BL	66.3 (20.5)				
Rudell 2016^c^ [57] Advanced NSCLC, EGFR+	2 L OSI at BL	65.2 (20.33)	EQ-5D-5 L VAS	N/A	Multinational patients with EGFR+ advanced NSCLC, 2 L after previous TKI	No
	2 L OSI at 36 weeks	73.7 (17.33)				
Schuette 2012 [51] NSCLC Stage IIIB–IV	2 L, PEM at BL	0.66 (0.256)	EQ-5D	UK TTO	Austrian and German advanced/mNSCLC 2 L patients mainly after prior platinum treatment (IIIa, 6.7%; IIIb, 19.8%; IV, 73.5%)	Meets NICE/SMC requirements
	2 L, PEM at 6 weeks (2nd cycle)	0.02 (0.214)	EQ-5D gain			
	2 L, PEM at 6th cycle	0.11 (0.228)				
	2 L, PEM at BL	59.3 (17.8)	EQ-5D VAS	N/A		No
	2 L, PEM at 6 weeks (2nd cycle)	3.3 (12.58)	EQ-5D VAS gain	N/A		
	2 L, PEM at 6th cycle	12.8 (17.62)				
Vargas 2009^c^ [72] Advanced NSCLC	2 L, on ERL	0.81	Global QoL index	NR	Patients with advanced NSCLC, 2 L after previous chemotherapy	No
	2 L, on taxanes	0.62				
≥ 2nd line						
Chen 2010^c^ [73] Advanced NSCLC^d^	2 L, DOC, during treatment	0.45^i^	SG	N/A	UK general public (as algorithm based on Nafees 2008 data used to calculate utilities)	Acceptable data for SMC
	2 L, DOC, after treatment	0.57				
	2 L, PEM, during treatment	0.54				
	2 L, PEM, after treatment	0.59				
	3 L, ERL, during treatment	0.48				
	BSC, during treatment	0.47				
Chevalier 2013 [38] Advanced/metastatic NSCLC	3/4 L PF	0.61 (0.3)	EQ-5D	French (TTO)	Stage I–Stage III/IV PF and PD	Meets HAS requirements
	3/4 L PD	0.42 (0.40)				
Griebsch 2014 [37] Stage IIIb (with pleural effusion)/IV NSCLC adenocarcinoma (LUX-LUNG 1)^j^	Week 4, progression effect longitudinal model	−0.1	EQ-5D	UK	Multinational advanced/metastatic NSCLC, 2 LL	Meets NICE requirements
	Mixed effect longitudinal model IRC	−0.056 (95% CI, − 0.083 to − 0.028)				
	Mixed effect longitudinal model IN	−0.065 (95% CI, − 0.092 to − 0.039)				
	Mixed effect longitudinal model IRC, AFA	−0.06				
	Mixed effect longitudinal model IRC, BSC	−0.046				
	Mixed effect longitudinal model IINV, AFA	−0.081				
	Mixed effect longitudinal model IINV, BSC	−0.033				
	Week 4, progression effect longitudinal model	−7.3	EQ-5D VAS	N/A		No
	Mixed effect longitudinal model IRC	−3.76 (95% CI, −5.19 to −2.32)				
	Mixed effect longitudinal model INV	− 3.83 (95% CI, − 5.21 to − 2.44)				
	Mixed effect longitudinal model IRC, AFA	3.63				
	Mixed effect longitudinal model IRC, BSC	−4.11				
	Mixed effect longitudinal model INV, AFA	−4.42				
	Mixed effect longitudinal model INV, BSC	−2.55				
Hirsh 2013 [40] Stage IIIB/IV NSCLC	3 LL on AFA + BSC	0.71	EQ-5D	UK	98% adenocarcinoma PD following treatment lines 1–2, one of which was platinum based, plus PD after at least 12 weeks of ERL or GEF	Meets NICE requirements
	3 LL on PLA + BSC	0.67				
	3 LL on AFA + BSC	67.4	EQ-5D VAS	N/A		No
	3 LL on PLA + BSC	65.2				
Schwartzberg^c^ 2015 [60] Stage IIIb/IV NSCLC (SQ & NSQ)	All patients wk 6	1.0 (21.7)	EQ-5D VAS	N/A	Patients, 2 LL, NIVO 3 mg/kg i.v. q2w	No
	wk 12	5.8 (21.3)				
	wk 18	8.2 (22.3)				
	wk 24	8.2 (23.9)				
	wk 30	8.4 (29.2)				
	SDis wk 6	3.8 (19.8)				
	wk 12	6.4 (21.9)				
	wk 18	8.2 (20.9)				
	wk 24	5.2(21.9)				
	wk 30	7.2 (28.5)				
	PR wk 6	7.3 (22.4)				
	wk 12	6.6 (24.7)				
	wk 18	8.1 (27.6)				
	wk 24	18.1 (31.0)				
	wk 30	13.7 (38.2)				
	PD wk 6	−5.8 (21.1)				
	wk 12	−3.0 (19.8)				
	wk 18	3.9 (24.3)				
	wk 24	6.8 (12.2)				
	wk 30	5.5 (15.7)				
Treatment line not specified						
Bradbury 2008^c^ [42] Advanced NSCLC	On ERL	0.772	EQ-5D	NR (possibly Canadian)	Canadian patients	Potentially relevant to CADTH
	On BSC	0.754				
Chang 2016^c^ [63] Advanced NSCLC	> 360 days from death	0.904 (95% CI, 0.892–0.917)	TTO	NR	General public, South Korea	No
	180–360 days from death	0.720 (95% CI, 0.692–0.748)				
	90–180 days from death	0.627 (95% CI, 0.598–0.655)				
	30–90 days from death	0.379 (95% CI, 0.349–0.409)				
	< 30 days from death	0.195 (95% CI, 0.172–0.218)				
Dansk 2016^c^ [43] Advanced NSCLC	Synthesized PF	Median, 0.706 Range, 0.620–0.815	Synthesized utility across > 1 instrument type	NR	Utilities synthesized included those where respondents were patients and those where they were the general public considering a hypothetical health state	No
	Synthesized PF trial-based	Median, 0.750 Range, 0.627–0.815				
	Synthesized PF non-trial-based	Median, 0.653 Range, 0.620–0.653				
	Synthesized PD	Median, 0.565 Range, 0.470–0.688				
	Synthesized PD trial-based	Median, 0.599 Range, 0.550–0.688				
	Synthesized PD non-trial-based	Median, 0.473 Range, 0.470–0.530				
Doyle 2008 [65] Metastatic NSCLC	SDis, no additional symptoms	0.626	SG	N/A	General public	No
	Treatment response, no additional symptoms	0.712				
Grunberg 2009^c^ [58] BC/LC	Chemotherapy-induced nausea and vomiting of differing severity	Reported graphically	SG	N/A	Patients BC/LC	Meets NICE requirements
Grutters 2010^c^ [44] NSCLC (stage unspecified)	NSCLC with grade 3+ dyspnoea, stage unspecified	Median, 0.52	EQ-5D-5 L	NR	Patients at an early treatment stage	No
Jang 2010 [47] Stage IV NSCLC	Stage IV NSCLC	0.75 (0.15)	EQ-5D	US	Patients with NSCLC attending a major Canadian cancer center outpatient clinic	No
Linnet 2015^c^ [62] Stage IV NSCLC on oral VINO	PCS, cycle 2	37.0	SF-12	N/A	Patients	No
	PCS, cycle 3	38.6				
	MCS, cycle 2	47.7				
	MCS, cycle 3	44.2				
	PCS, cycle 2	52.9			Caregivers	Potential to estimate SF-6D for caregivers to mNSCLC patients, for SMC or CADTH
	PCS, cycle 3	53.4				
	MCS, cycle 2	46.2				
	MCS, cycle 3	44.6				
Lloyd 2005^c^ [66] Stage IV NSCLC	RES	0.70	SG^k^	N/A	General public	No
	SDis, oral treatment	0.63				
	SDis, i.v. treatment	0.58				
	PD	0.42				
	End of life	0.33				
Manser 2006 [61] Stage IV NSCLC	Stage IV	Median, 0.68 (IQR, 0.54–0.82)	AQoL	Australia	Mixed stage enrolled: I, 31.5%; II, 17.4%; IIIa, 16.3%; IIIb, 7.6%; IV, 25.0%	No
Matza 2014 [67] Stage IV cancer with bone metastases	Cancer with bone metastases and no SRE	0.47 (0.41)	TTO	N/A	General public, UK (Edinburgh and London)	No
		0.47 (0.45)			General public, Canada (Montreal and Toronto)	
		0.47 (0.42)			General public, UK and Canada	
Stewart 2015 [56] EGFR+ Stage IV NSCLC	PR/SDis on EGFR TKIs (GEF, ERL, AZD9291)	0.82 (SE, 0.16)	EQ-5D-3 L	NR	Patients, eligible for or on TKI tx, 55% Asian, 45% male, median age 60, 66% never smokers. Stage IV: at diagnosis, 80% when surveyed, 100%	Unclear
	Responded to standard chemotherapy	0.80 (SE, 0.12)				
	EGFR+, responded to GEF	0.84 (SE, 0.14)				
	EGFR+, responded to ERL	0.82 (SE, 0.17)				
	EGFR+, responded to AZD9291	0.83 (SE, 0.16)				
	EGFR+, PD during TKI treatment (GEF, ERL, AZD9291)	0.74 (SE, 0.08)				
	EGFR+, all patients (PR/SDis/PD), 25% 3LL	0.802				
Tabberer 2006 [52] Advanced NSCLC	RES	0.49	EQ-5D	NR	General public, UK (Cardiff, Glasgow, London and Oxford)	No
	SDis	0.46				
	SDis + oral treatment	0.45				
	SDis + i.v. treatment	0.43				
	PD	0.22				
	Near death	0.15				
Trippoli 2001 [53] Metastatic NSCLC	Metastatic NSCLC	0.53 (0.36)	EQ-5D	UK (TTO)	Italian patients	Meets NICE and SMC reference cases
		0.55 (0.22)^l^	EQ-5D VAS	N/A		No
Yang 2014 [54] Stage IIIB/IV NSCLC	Stage IV inoperable, performance status 0–1	0.75 (0.22)	EQ-5D	Taiwan	Patients, mixed NSCLC stages: I, 0.8%; II, 0%; IIIA, 4.5%; IIIB, 16.9%; IV, 77.8%	No
	Stage IV inoperable, performance status 0–4	0.75 (0.22)				
Yokoyama 2013^c^ [55] Advanced NSCLC/SCLC	Stage IIIB/IV NSCLC/SCLC with bone metastasis and SRE	NR	EQ-5D	NR	Patients, advanced NSCLC, 72%, SCLC, 28% NSCLC and SCLC: IIIB, 37%; IV, 63%	No

^a^Mean, or mean (SD) unless stated otherwise

^b^VAS scores were also reported in this study but unclear whether this was EQ-VAS

^c^These studies were published as abstracts or posters

^d^This referenced article (https://www.ncbi.nlm.nih.gov/pubmed/10109801) is for a VAS valuation

^e^ *SDis vignette:*

• You have a life-threatening illness that is stable on treatment. You are receiving cycles of treatment that require you to go to the outpatient clinic

• You have lost weight, and your appetite is reduced. You sometimes experience pain or discomfort in your chest or under your ribs, which can be treated with painkillers. You have shortness of breath, and breathing can be painful. You have a persistent nagging cough

• You are able to wash and dress yourself and do jobs around the home. Shopping and daily activities take more effort than usual

• You are able to visit family and friends but often have to cut it short because you get tired

• You sometimes feel less physically attractive than you used to. Your illness has affected your sex drive

• You worry about dying and how your loved ones will cope

^f^ *Second-line responding vignette:*

• You have a life-threatening illness that is responding to treatment. You are receiving cycles of treatment which require you to go to the outpatient clinic

• You are gaining back your weight and your appetite is returning. You occasionally experience pain or discomfort in your chest or under your ribs which can be treated with painkillers. You sometimes have shortness of breath. You occasionally have a nagging cough

• You are able to wash and dress yourself and do jobs around the home. Shopping and daily activities can sometimes be tiring

• You are able to visit family and friends but sometimes have to cut it short because you get tired

• You occasionally feel less physically attractive than you used to. Your illness has somewhat affected your sex drive

• You sometimes worry about dying and how your loved ones will cope

^g^ *Second-line PD vignette:*

• You have a life-threatening illness, and your condition is getting worse

• You have lost your appetite and have experienced significant weight loss. You experience pain and discomfort in your chest or under your ribs. You frequently have shortness of breath, and breathing is often painful. You have a persistent nagging cough and sometimes cough up blood. You may experience some difficulty swallowing

• You experience severe fatigue and feel too tired to go out or to see family and friends. It has affected your relationships with them

• You need assistance to wash and dress yourself. You are often unable to do jobs around the house or other daily activities. You are dependent on others to do your shopping and are unable to do your usual daily activities

• You often feel less physically attractive than you used to. You have little or no sexual drive

• You are depressed, and dying is always on your mind. You worry about how your loved ones will cope

^h^This study also has utilities available every 3 weeks between week 0 and week 30 for all treatments

^i^All utilities in this paper assumed to be the mean, although it is not clearly stated in the paper

^j^1 L data also reported from LUX-LUNG 3

^k^Individual country values are also available in this publication

^l^This value is reported as in the original publication

*Abbreviations: 1 L* first line, *2 L* second line, *2 LL* second and subsequent line, *3 LL* third and subsequent line, *AFA* afatinib, *ALK+* anaplastic lymphoma kinase mutation positive, *AQoL* Assessment of Quality of Life instrument, *BC* breast cancer, *BL* baseline, *BSC* best supportive care, *CADTH* Canadian Agency for Drugs and Technologies in Health, *CI* confidence interval, *CRZ* crizotinib, *DOC* docetaxel, *EGFR* epidermal growth factor receptor, *EGFR+* epidermal growth factor receptor mutation positive, *EORTC QLQ* European Organisation for the Research and Treatment of Cancer Quality of Life Questionnaire, *EQ-VAS* EuroQol visual analogue scale, *ERL* erlotinib, *GEF* gefitinib, *HAS* Haute Autorité de Santé, *HTA* health technology assessment, *IQR* interquartile range, *i.v.* intravenous, *LC* lung cancer, *MCS* mental component summary, *mNSCLC* metastatic non-small cell lung cancer, *N/A* not applicable, *NICE* National Institute for Health and Care Excellence, *NIN* nintedanib, *NIVO* nivolumab, *NR* not reported, *NSCLC* non-small cell lung cancer, *NSQ* non-squamous, *NTS* not treatment-specific, *OSC* optimal standard care, *OSI* osimertinib, *PCS* physical component summary, *PD* progressive disease, *PEM* pemetrexed, *PF* progression-free, *PLA* placebo, *PR* partial response, *PSS* progression-status specific, *q2w* once every 2 weeks, *QoL* quality of life, *RES* response, *SCLC* small-cell lung cancer, *SD* standard deviation, *SDis* stable disease, *SE* standard error, *SF-6D* 6-dimension Short-Form Health Survey, *SF-12/36* 12-/36-item Short-Form Health Survey, *SG* standard gamble, *SMC* Scottish Medicines Consortium, *SQ* squamous, *SRE* skeletal-related event, *TKI* tyrosine kinase inhibitor, *TTO* time trade-off, *VAS* visual analogue scale, *VINO* vinorelbine, *WBRT* whole-brain radiotherapy

Disutilities for progression from a stable state were − 0.056 or − 0.065 by EQ-5D, both from Griebsch et al. [37], or − 0.1798 by general population-derived SG [69]. Overall, HSUVs varied not only by treatment line and disease state, but also by the treatment received under the same health state (potentially reflecting differences in safety profiles) and by the instrument/tariff used to derive the HSUV.

### Relevant disutilities and decrements

Eleven studies identified in this systematic review reported disutilities or decrements for AE health states [44, 52, 55, 58, 59, 65, 67–71]. Only two studies reported disutilities specifically associated with second-line treatment [69, 71], and another two studies did not specify the treatment line [44, 65]; disutility and decrement data are summarized in Table 4. Utility-incorporating decrements were identified for the following AEs in the context of second-line “stable disease” or second-line “responding”: diarrhoea, fatigue, febrile neutropenia, hair loss and nausea/vomiting. Disutilities associated with second-line treatment were reported for the following events [69]: “moving from stable to progressive state” (− 0.18), neutropenia (− 0.09), febrile neutropenia (− 0.09), fatigue (− 0.07), nausea/vomiting (− 0.05), diarrhoea (− 0.05), hair loss (− 0.04) and rash (− 0.03).

**Table 4:** Disutilities and decrements for adverse event health states in patients with previously treated mNSCLC

Relevance	Author, year, country, reference^a^	Instrument and respondent	Utility type	Health state/disutility	Mean HSUV (SD) [SE] {95% CI}		HTA suitability
Advanced/mNSCLC	Nafees 2008, UK [69]	SG General public	UID	Stage IV NSCLC, 2 L, stable disease			Does not meet HTA body reference case as vignette-based utility completed by general public respondents. Has been used in multiple HTA submissions, however. Specifically 2 L and UK, good sample size (*n*=100) and measure of dispersion available.
				+ Diarrhoea	0.61		
				+ Fatigue	0.58		
				+ Febrile neutropenia	0.56		
				+ Hair loss	0.61		
				+ Nausea/vomiting	0.61		
				+ Neutropenia	0.56		
				+ Rash	0.62		
				Stage IV NSCLC, 2 L, responding disease			
				+ Diarrhoea	0.63		
				+ Fatigue	0.60		
				+ Febrile neutropenia	0.58		
				+ Hair loss	0.63		
				+ Nausea/vomiting	0.62		
				+ Neutropenia	0.58		
				+ Rash	0.64		
			D	Stage IV NSCLC, 2 L, moving from stable to progressive	−0.18 [0.022]		
				Neutropenia	−0.09 [0.015]		
				Febrile neutropenia	−0.09 [0.016]		
				Fatigue	−0.07 [0.018]		
				Nausea and vomiting	−0.05 [0.016]		
				Diarrhoea	−0.05 [0.016]		
				Hair loss	−0.04 [0.015]		
				Rash	−0.03 [0.012]		
				Response gain	0.02 [0.007]		
	Tabberer 2006, UK [52]	EQ-5D (tariff NR but likely UK TTO tariff as UK sample) General public	D	Compared with stable disease (advanced NSCLC, line not specified)			Not suitable as general public respondents, line of treatment not specified, and no measure of dispersion reported. Good sample size, however (*n*=154) and Nafees et al. 2008 in 2 L does not provide disutilities for neuropathy or stomatitis so these values from Tabberer et al. may be the best available.
				Febrile neutropenia	−0.27		
				Rash	−0.06		
				Neuropathy	−0.15		
				Neutropenia	−0.14		
				Nausea	−0.14		
				Stomatitis	−0.14		
				Diarrhoea	−0.13		
	Doyle 2008, UK [65]	SG General public	UID	Metastatic NSCLC, line not specified, SDis no additional symptoms			Does not meet reference case as general public respondents. However, these are the only disutilities for severe symptoms for cough, dyspnoea and pain in mNSCLC, so are best option in spite of not meeting HTA derivation method preferences (*n*=101)
				+ Cough	0.58		
				+ Dyspnoea	0.58		
				+ Pain	0.56		
				+ Cough, dyspnoea and pain	0.46		
			D	Cough	−0.05 [0.011]		
				Dyspnoea	−0.05 [0.012]		
				Pain	−0.07 [0.012]		
				Cough, dyspnoea and pain^c^	*− 0.17* ^b^		
				Responding disease gain vs SDis	0.09 [0.015]		As line not specified, data from Nafees et al. 2008 should be used in preference.
	Handorf 2012, USA [70]	Expert opinion	UID	Stage IV NSCLC adenocarcinoma (1 L SDis)			
				+ neutropenia	0.67		Does not meet reference case as expert opinion-derived. This AE is covered by Nafees et al. 2008, which uses a better derivation method than expert opinion.
				+ pneumothorax	0.63		Does not meet reference case as expert opinion-derived, but these are the only estimates for these AE health states. SDis estimate was 0.670 (oral therapy) and 0.653 (i.v. chemotherapy) for disutility calculation.
				+ haemorrhage	0.63		
				+ thrombocytopenia	0.65		
				+ thrombosis	0.56		
Earlier stage NSCLC (curative intent)	Grutters 2010, country NR [44]	EQ-5D-5 L (tariff NR) Patients	UID	NSCLC, curative intent stage, line not specified, grade III+ dyspnoea	0.52 (median)		Patient-derived EQ-5D but tariff and measure of dispersion NR. Only source of grade III+ dyspnea. Utility for NSCLC patients without dyspnoea in this sample was 0.81, i.e. disutility − 0.29
Advanced/mLC (NSCLC+SCLC)	Yokoyama 2013, Japan [55]	EQ-5D (tariff NR) Patients	D	Stage IIIB/IV NSCLC/SCLC with bone metastases + skeletal related event (pathologic fracture, radiation or surgery to bone lesion, spinal cord compression or hypercalcaemia)	− 0.05^d^		Provides NSCLC/SCLC (mixed) patient-derived EQ-5D decrement for (mixed) SREs. Data for these AEs are limited, so although this estimate is not robust (n=9 and response % low at 32%) it does provide an indication. No variability measure reported
Breast cancer and lung cancer	Grunberg 2009, USA [58]	SG Patients	UID	Base state: continuous nausea and vomiting	0.53^f^		Nafees et al. 2008 provide data for nausea and vomiting but if different levels of nausea and vomiting need to be discerned then these utilities can be considered. Patient-derived SG but mixed lung/breast cancers. Good sample size (*n*=96) but no measure of dispersion. As the Copyright fee for Grunberg 2009 was high, these data are reported from Shabaruddin 2013 [79] (a previous SR that extracted the graphical data from Grunberg 2009)
			Increment	Limited nausea and limit vomiting vs continuous nausea and vomiting	+ 0.53^f^		
			Increment	Limited nausea vs continuous nausea and vomiting	+ 0.55^f^		
			Increment	Limited vomiting vs continuous nausea and vomiting	+ 0.50^f^		
Advanced Cancer	Matza 2014, UK [67]	TTO General public	U	Stage IV cancer with bone metastases (no skeletal-related events)	0.47 (0.41)		Does not meet reference case as general population respondents. However, as there are no alternative utilities for bone metastases these UK utilities could be considered for NICE or SMC. Good sample size (*n*=126), SD available.
			UID	+ spinal cord compression without paralysis	0.25 (0.50)		
				+ spinal cord compression with paralysis	0.13 (0.49)		
				+ fracture of the leg	0.42 (0.41)		
				+ fracture of the rib	0.44 (0.42)		
				+ fracture of the arm	0.44 (0.41)		
				+ radiation treatment (2 weeks, 5 appointments/week)	0.42 (0.42)		
				+ radiation treatment (2 appointments)	0.45 (0.41)		
				+ surgery to stabilize bone	0.40 (0.44)		
	Matza 2014, Canada [67]	TTO General public	U	Stage IV cancer with bone metastases (no skeletal-related events)	0.47 (0.45)		Does not meet reference case as general population respondents. However, as there are no alternative utilities for bone metastases these Canadian utilities could be considered for CADTH. Reasonable sample size (*n*=61), SD available.
			UID	+ spinal cord compression without paralysis	0.25 (0.54)		
				+ spinal cord compression with paralysis	0.19 (0.53)		
				+ fracture of the leg	0.40 (0.48)		
				+ fracture of the rib	0.43 (0.47)		
				+ fracture of the arm	0.43 (0.48)		
				+ radiation treatment (2 weeks, 5 appointments/week)	0.41 (0.50)		
				+ radiation treatment (2 appointments)	0.45 (0.45)		
				+ surgery to stabilize bone	0.39 (0.50)		
	Matza 2014, UK and Canada [67]	TTO General public	U	Stage IV cancer with bone metastases (no skeletal-related events)	0.47 (0.42)		Does not meet reference case as general population respondents. However, as there are no alternative utilities for bone metastases these UK+Canadian utilities could be considered for NICE, SMC or CADTH. Good sample size (*n*=187), SD available.
			UID	+ spinal cord compression without paralysis	0.25 (0.21)		
				+ spinal cord compression with paralysis	0.15 (0.50)		
				+ fracture of the leg	0.41 (0.43)		
				+ fracture of the rib	0.44 (0.43)		
				+ fracture of the arm	0.43 (0.43)		
				+ radiation treatment (2 weeks, 5 appointments/week)	0.41 (0.45)		
				+ radiation treatment (2 appointments)	0.45 (0.42)		
				+ surgery to stabilize bone	0.40 (0.46)		
	Matza 2014, UK [67]	TTO General public	D	Stage IV cancer with bone metastases			As above
				+ spinal cord compression without paralysis	−0.22 (0.31)		
				+ spinal cord compression with paralysis	−0.34 (0.36)		
				+ fracture of the leg	−0.05 (0.09)		
				+ fracture of the rib	−0.03 (0.08)		
				+ fracture of the arm	−0.03 (0.07)		
				+ radiation treatment (2 weeks, 5 appointments/week)	−0.05 (0.12)		
				+ radiation treatment (2 appointments)	−0.02 (0.07)		
				+ surgery to stabilize bone	−0.07 (0.17)		
	Matza 2014, Canada [67]	TTO General public	D	Stage IV cancer with bone metastases			As above
				+ spinal cord compression without paralysis	−0.22 (0.32)		
				+ spinal cord compression with paralysis	−0.28 (0.30)		
				+ fracture of the leg	−0.07 (0.19)		
				+ fracture of the rib	−0.04 (0.17)		
				+ fracture of the arm	−0.04 (0.07)		
				+ radiation treatment (2 weeks, 5 appointments/week)	−0.06 (0.21)		
				+ radiation treatment (2 appointments)	−0.02 (0.11)		
				+ surgery to stabilize bone	−0.08 (0.21)		
	Matza 2014, UK and Canada [67]	TTO General public	D	Stage IV cancer with bone metastases			As above
				+ spinal cord compression without paralysis	−0.22 (0.31)		
				+ spinal cord compression with paralysis	−0.32 (0.34)		
				+ fracture of the leg	−0.06 (0.13)		
				+ fracture of the rib	−0.03 (0.12)		
				+ fracture of the arm	−0.04 (0.11)		
				+ radiation treatment (2 weeks, 5 appointments/week)	−0.06 (0.15)		
				+ radiation treatment (2 appointments)	−0.02 (0.08)		
				+ surgery to stabilize bone	−0.07 (0.18)		
Cancer, unclear stage	Lloyd 2008, UK [59]	SG General public	UID	Anaemia associated with cancer treatment			Does not meet reference case as general population sample respondent for SG exercise.
				Haemoglobin level (g/dL)	7.0–8.0	0.58 {0.067}	
					8.0–9.0	0.61 {0.064}	
					9.0–10.0	0.64 {0.060}	
					10.0–10.5	0.64 {0.062}	
					10.5–11.0	0.66 {0.061}	
					11.0–12.0	0.70 {0.056}	
					>12.0	0.71 {0.057}	
		VAS General public	UID	Haemoglobin level (g/dL)	7.0–8.0	16.9 {2.6}	
					8.0–9.0	22.3 {3.0}	
					9.0–10.0	27.6 {2.9}	
					10.0–10.5	32.9 {3.4}	
					10.5–11.0	38.8 {3.6}	
					11.0–12.0	45.9 {4.2}	
					>12.0	51.2 {4.3}	
		TTO Cancer patients with recent experience of chemotherapy-related anaemia or fatigue	UID	Haemoglobin level (g/dL)	7.0–8.0	0.30 {0.127}	
					8.0–9.0	0.36 {0.126}	
					9.0–10.0	0.41 {0.125}	
					10.0–10.5	0.45 {0.122}	
					10.5–11.0	0.45 {0.111}	
					11.0–12.0	0.55 {0.105}	
					>12.0	0.61 {0.112}	
			U	Own current health	0.85 {0.034}		
				EQ-5D current health	0.87 {0.076}		
		VAS Cancer patients with recent experience of chemotherapy-related anaemia or fatigue	UID	Haemoglobin level (g/dL)	7.0–8.0	21.7 {5.7}	
					8.0–9.0	32.4 {6.6}	
					9.0–10.0	34.2 {6.7}	
					10.0–10.5	41.9 {6.6}	
					10.5–11.0	44.7 {6.6}	
					11.0–12.0	52.2 {6.8}	
					>12.0	62.4 {7.9}	
			U	Own current health	87.6 {4.9}		
				EQ-5D current health	84.2 {4.57}		
NR	Westwood 2014, NR [71]	NR	D	Anaemia	0.073 [0.018]		Disutilities for anaemia and treatment mode have been used in previous NICE submissions. However, there is no information concerning their derivation.
2 L NSCLC				Oral therapy (ERL)	0.014 [0.012]		
2 L NSCLC				i.v. therapy	0.043 [0.020]		
Patients without NSCLC 1 L setting	Nafees 2016, Multinational and UK [68]^g^	TTO Patients (but not NSCLC patients) from the general public in UK, Australia, France, China, S. Korea, Taiwan	D	Bleeding vs BL (stable no side effects)	−0.25		No
				Hypertension vs BL (stable no side effects)	−0.03		
			UID	Responding + bleeding vs BL	0.534		
				Responding + hypertension vs BL	0.749		
				Stable + bleeding vs BL	0.508		
				Stable + hypertension vs BL	0.729		

^a^Some studies identified in this systematic review were not included in this table for the following reasons: Grunberg 2009 [58] and Grutters 2010 [44] did not report values

^b^The italics indicate a calculated utility. This is calculated from the values reported in Doyle 2008 [65] Table 3 (difference between stable disease no other symptoms and stable disease with cough, dyspnoea and pain, to obtain the disutility). It is not calculated by adding the disutilities, as this would not be valid

^c^Values were calculated from ‘SDis + cough, dyspnea and pain’ utility minus ‘SDis no additional symptoms’ utility

^d^The study did not indicate if mean or median

^e^Some studies identified in this systematic review were not included in this table for the following reasons: Grunberg 2009 [58] and Grutters 2010 [44] did not report values, and Nafees 2016 [68] reported variation in two decrements (bleeding, hypertension) based on the different populations in which valuation was undertaken

^f^As reported in Shabarruddin 2013 [79], base state and utility increments were presented on different scales: base state was based on standard gamble scale between perfect health (arbitrary score of 100) or immediate death (arbitrary score of 0) while the utility increments were based on a scale between perfect health (arbitrary score of 100) and the surrogate negative anchor of continuous nausea/vomiting (re-set to an arbitrary score of 0)

^g^Values are presented for global population (United Kingdom, Australia, France, China, Taiwan, Korea). Note that country-specific data are also available

*Abbreviations: 1 L* first line, *BL* baseline, *CI* confidence interval, *D* decrement, *ERL* erlotinib, *HSUV* health state utility value, *HTA* health technology assessment, *i.v.* intravenous, *LC* lung cancer, *mLC* metastatic lung cancer, *mNSCLC* metastatic non-small cell lung cancer, *NICE* National Institute for Health and Care Excellence, *NR* not reported, *NSCLC* non-small cell lung cancer, *SCLC* small cell lung cancer, *SD* standard deviation, *SE* standard error, *SG* standard gamble, *TTO* time trade-off, *U* utility, *UID* utility incorporating decrement for adverse events, *VAS* visual analogue scale

Further recommended sources of AE health state (dis) utilities were as follows (Fig. 1): in 2 L from general population SG in Nafees et al. 2008 [69]; in metastatic NSCLC (line unspecified) from general population SG in Doyle et al. 2008 [65]; in 1 L from patients without NSCLC using directly elicited TTO in Nafees et al. 2016 [68]; in 2 L in NSCLC as reported in Westwood et al. 2014 [71]; in cancer with bone metastases for skeletal-related events from general population TTO in Matza et al. 2014 [67]; stage IV NSCLC in 1 L from expert opinion estimates in Handorf et al. 2012 (expert-opinion-derived utilities from this study were included, as they are the only source of estimates for pneumothorax, thrombocytopenia and thrombosis disutilities) [70]; and anaemia from general population SG or from patient-derived TTO in Lloyd et al. 2008 [59].

### Description of HTA-relevant HSUVs and disutilities

Of the 36 publications, 13 provided HSUVs that meet the NICE reference case or are considered acceptable to the HTA agencies of interest [37–40, 42, 45, 46, 49, 53, 56, 58, 64, 69], based on the measurement technique for generation of HSUVs, as outlined in Additional file 3: Figure S1. The main characteristics of these studies are presented in Table 3. These 13 publications reported data from multinational studies [37–40, 45, 49, 64], and from Canada [42, 56], France/Germany [46], USA [58], Italy [53] and the UK [69]. In these studies, HRQoL was measured using EQ-5D [37–40, 42, 45, 46, 49, 53, 56], EQ-VAS [37, 39, 40, 49] and SG [58, 64, 69]. The HTA suitability of disutilities and decrements for AE health states in previously treated patients are reported in Table 4.

## Discussion

Economic evaluation, particularly cost–utility analysis, provides important information for guiding decision-making in health care, and its use in HTA is increasing globally. Such evaluation includes examination of the time spent in different disease states and uses an HSUV for each disease state to calculate QALYs; HSUVs therefore play a key role in economic evaluation. As summarized in Additional file 3: Figure S1, NICE, SMC, CADTH, HAS and PBAC prefer utilities to be estimated using a generic preference-based instrument, with health states described by patients through use of a questionnaire, and with the health state valued using a country-specific tariff that reflects societal preferences. As the aim of this systematic review was to evaluate the experience of adults with previously treated mNSCLC, the synthesis of health state utility estimates was outside its scope. However, the findings presented here may provide a basis for generation of an accurate estimate of the mean HSUV for use in economic evaluations [74, 75].

This systematic review identified HSUVs relevant to the experience of previously treated adult patients with mNSCLC. Search strings were designed to allow (dis) utilities from a broader population (including lung cancer, advanced/metastatic cancer and specific metastases common in patients with lung cancer). In the absence of second-line mNSCLC (dis) utilities, alternatives were selected with decreasing population specificity and relevance from first-line mNSCLC, NSCLC, lung cancer or advanced/metastatic cancer, as outlined in Fig. 1. Ordering the HSUVs by line of treatment reflects the practice of switching treatment at progression. However, for the newer immunotherapies, patients may remain on treatment post-progression, and their HRQoL may remain at pre-progression levels. Thus, HSUVs estimated for progression status-specific health states from patients receiving chemotherapy may not be suitable to apply to the equivalent health states when patients receive immunotherapy.

In total, the 36 identified articles reported 591 HSUVs relevant to the experience of previously treated adult patients with mNSCLC, and 11 of these studies reported a total of 195 disutilities or decrements for AE health states that are relevant to the experience of patients with mNSCLC. The range of HSUVs identified for comparable health states, such as progression-free/stable disease among patients treated second-line for advanced/metastatic NSCLC [39, 45], highlights how differences in study type, tariff, health state and the measures used can drive variation in HSUV estimates. For instance, disutilities for progression from a stable state were − 0.056 or − 0.065 using EQ-5D, [37] or − 0.1798 by general-population-derived SG. [69] To overcome such variations, where possible, HSUV studies should seek to use instruments, respondents and valuation populations that are most acceptable to HTA bodies. However, there are instances where variation in methods can be justified. For example, disutility values derived from vignettes and a general public sample were used by Nafees et al. [69], because asking patients suffering such toxicities to complete HRQoL questionnaires was considered to be too burdensome for patients and potentially unethical. Moreover, although the variation may be large, it helps decision makers to identify where variability exists and informs the design of sensitivity analyses.

In the 36 publications identified, 13 provided HSUVs that meet the NICE reference case or are considered acceptable to the HTA agencies of interest [37–40, 42, 45, 46, 49, 53, 56, 58, 64, 69]. These were deemed suitable because HRQoL was measured using the EQ-5D [37–40, 42, 45, 46, 49, 53, 56] or SG [58, 64, 69], both measures preferred or accepted by several HTA authorities. This endeavour fills an important gap in the field because hitherto, only two reports had described HSUVs in mNSCLC [68, 69]; neither was a systematic review of the literature, nor did they assess their appropriateness for use in economic evaluations.

This systematic review did not identify an HSUV report based on data from the OAK trial (NCT02008227), because it was published as a congress abstract after the cut-off date for literature searching [76]. However, the HSUVs are relevant to the aims of this systematic review, and a brief description is provided below for completeness. Patients with locally advanced NSCLC or mNSCLC after failure of platinum-containing chemotherapy were randomized in a phase 3 trial to receive atezolizumab or docetaxel [76, 77]. As part of the trial, patients completed the EQ-5D, and the resultant HSUVs were presented by time point before death. This study is similar to Huang et al. 2016, which also presented time-to-death EQ-5D utilities for a similar patient group receiving immunotherapy, except comparing pembrolizumab and docetaxel [45]. Overall, HSUVs were very similar between studies at approximately equivalent time points. In the OAK study, the following HSUVs were reported by time point before death: 0.77 (> 210 days), 0.71 (105–210 days), 0.61 (35–105 days) and 0.39 (< 35 days). For comparison, HSUVs published by Huang et al. 2016 were 0.73 (180–360 days), 0.69 (90–180 days), 0.60 (30–90 days) and 0.40 (< 30 days). A further study evaluating the efficacy of immunotherapy in patients with NSCLC showed that baseline mean EQ-VAS and EQ-5D index scores were similar for nivolumab (63.7 and 0.68, respectively) and docetaxel (66.3 and 0.66, respectively) [50].

Strengths of this systematic review include the wide range of data sources searched and the search string design, which enabled identification of disutilities and utility decrements for a wide range of AEs and progressive disease states (e.g. common sites of metastasis from lung cancer) of relevance to the experience of patients previously treated for mNSCLC. We have presented HSUVs by line of treatment, allowing use in economic modelling, and have discussed HSUVs likely to be accepted by the HTA bodies of interest. Inadequate or inconsistent reporting is common, and low sample sizes and response rates considerably impact on the reported confidence intervals of the reported results. However, among the studies identified here, most reported sample size (over 100 respondents in most cases), many provided a measure of variability for the values reported, and several were based on response rates greater than 80% (although response rates were unreported in more than half of the studies). Moreover, the use of only published HSUVs can be a limitation, as HTA submissions may use HSUVs that have not been previously published. As part of this systematic review, we have therefore searched HTA submissions for any relevant utilities; most HTAs use data reported by Nafees et al. [69]

Limitations of this review include that the label for the upper bound of the utility scale (e.g. “full health” or “perfect health”) was not recorded. This has been shown to be a significant predictor of utility in lung cancer [78], so variation in utilities due to a different upper bound label cannot be explored. A further limitation concerns data extraction from some studies presented as congress abstracts or posters. Owing to the word restrictions placed on conference proceedings they may not be considered a robust data source in comparison with full publications. Furthermore, both screening and data extraction were conducted primarily by a single reviewer, and only 50% of studies were checked by a second reviewer. The exclusion of studies that used mapping to derive EQ-5D and utility values is a further limitation of this study; however, sufficient data obtained through direct measurement were identified to be informative.

## Conclusions

This systematic review begins to address the challenge of identifying reliable estimates of utility values in mNSCLC that are suitable for use in economic evaluations. Our work has also highlighted that these estimates are vulnerable to variations in study type, tariff, health state and the measures used, and that shortcomings in reporting are common.

## Additional files

Additional file 1: **Table S1.** Search strings. (DOCX 90 kb)

Additional file 2: **Table S2.** Listing of first-line mNSCLC studies with utility data excluded at second pass [80–100]. (DOCX 84 kb)

Additional file 3: **Figure S1.** Hierarchy of preferred methodology for generation of HSUVs for different HTA agencies. (PDF 1172 kb)

Additional file 4: **Table S3.** Quality assessment of identified studies. (DOCX 111 kb)

## Abbreviations

AE: Adverse event
AQoL: Assessment of Quality of Life instrument
CADTH: Canadian Agency for Drugs and Technologies in Health
HAS: Haute Autorité de Santé
HRQoL: Health-related quality of life
HSUV: Health state utility value
HTA: Health technology assessment
mNSCLC: Metastatic non-small cell lung cancer
NICE: National Institute for Health and Care Excellence
NSCLC: Non-small cell lung cancer
PBAC: Australian Pharmaceutical Benefits Advisory Committee
PICOS: Patient, intervention, comparator, outcome, study
PRISMA: Preferred Reporting Items for Systematic Reviews and Meta-Analyses
QALY: Quality-adjusted life-year
RECIST: Response Evaluation Criteria in Solid Tumors
SF-12/SF-36: 12-/36-item Short-Form Health Survey
SF-6D: 6-dimension Short-Form Health Survey
SG: Standard gamble
SMC: Scottish Medicines Consortium
TTO: Time trade-off
VAS: Visual analogue scale
