# Supplementary material for: Systematic review of health state utility values in metastatic non-small cell lung cancer with a focus on previously treated patients
Source: Health Qual Life Outcomes. 2018 Sep 12;16:179. doi: 10.1186/s12955-018-0994-8 (PMC6134713; doi:10.1186/s12955-018-0994-8)
Supplement: Supplementary file 1 — Table S1. Search strings. (DOCX 90 kb) [file 12955_2018_994_MOESM1_ESM.docx]

# Additional File 1 – Table S1 Search strings

**Databases: Embase and MEDLINE**

Date search run: 7 September 2016

Hits: 1521

Utilities filter: adapted from Arber 2015 [30]

| **No.** | **Query** | **Results** |
| --- | --- | --- |
| #1 | 'non small cell lung cancer'/exp OR nsclc*:ab,ti OR mnsclc*:ab,ti OR ansclc*:ab,ti OR msqnsclc*:ab,ti OR 'non sq nsclc':ab,ti OR sqclc*:ab,ti OR 'ns nsclc':ab,ti OR nsnsclc*:ab,ti OR 'la nsclc':ab,ti OR lansclc*:ab,ti OR cpnpc*:ab,ti OR (lac NEAR/3 (lung OR adenocarcinoma)):ab,ti OR ((scc NEAR/3 'squamous cell carcinoma'):ab,ti AND lung:ab,ti) OR (non NEAR/3 small NEAR/3 cell NEAR/3 lung NEAR/3 (cancer* OR carcinoma*)):ab,ti OR (('non small' OR nonsmall) NEAR/3 lung NEAR/3 cell NEAR/3 (cancer* OR carcinoma*)):ab,ti OR (('non small' OR nonsmall) NEAR/3 cell NEAR/3 lung NEAR/3 (cancer* OR carcinoma*)):ab,ti OR (bronchial NEAR/3 ('non small' OR nonsmall) NEAR/3 cell NEAR/3 (cancer* OR carcinoma*)):ab,ti OR ('non small cell' NEAR/3 (lung OR bronchial OR pulmonary OR bronchopulmonary OR bronchus) NEAR/3 (cancer* OR carcinoma*)):ab,ti OR ('non small' NEAR/3 cell NEAR/3 (cancer* OR carcinoma*) NEAR/3 lung*):ab,ti OR (pulmonary NEAR/3 'non small cell' NEAR/3 (cancer* OR carcinoma*)):ab,ti OR (large NEAR/3 cell NEAR/3 lung NEAR/3 (cancer* OR carcinoma*)):ab,ti OR ((squamous OR nonsquamous OR 'non squamous') NEAR/5 (cell OR 'non small cell') NEAR/3 lung NEAR/3 (cancer* OR carcinoma*)):ab,ti OR (bronchus NEAR/3 squamous NEAR/3 cell NEAR/3 (cancer* OR carcinoma*)):ab,ti OR (lung NEAR/3 epidermoid NEAR/3 (cancer* OR carcinoma*)):ab,ti OR (lung NEAR/3 squamous NEAR/3 cell NEAR/3 (cancer* OR carcinoma*)):ab,ti OR (lung:ab,ti OR poumon:ab,ti AND (nsclc*:ab,ti OR cpnpc*:ab,ti OR 'non small':ab,ti OR nonsmall:ab,ti OR large:ab,ti OR squamous:ab,ti OR 'non squamous':ab,ti OR nonsquamous:ab,ti OR 'non à petites cellules':ab,ti) AND (neoplasm*:ab,ti OR cancer*:ab,ti OR carcinoma*:ab,ti OR adenocarcinoma*:ab,ti OR angiosarcoma*:ab,ti OR chrondosarcoma*:ab,ti OR sarcoma*:ab,ti OR teratoma*:ab,ti OR lymphoma*:ab,ti OR blastoma*:ab,ti OR microcytic*:ab,ti OR carcinogenesis:ab,ti OR tumour*:ab,ti OR tumor*:ab,ti OR metasta*:ab,ti OR métastasé:ab,ti OR métastatique:ab,ti OR avancé:ab,ti OR 'progression localisée':ab,ti)) OR ((adenocancer OR adenocarcinoma) NEAR/3 (lung OR pulmonary)):ab,ti OR (((cancer OR tumeur) NEAR/3 (poumon OR bronchique)):ab,ti AND ('non à petites cellules':ab,ti OR 'non-lié à de petites cellules':ab,ti)) | 137 230 |
| #2 | 'quality adjusted life year'/exp OR 'quality of life index'/exp OR 'short form 36'/exp OR (utility:ab,ti AND ('quality of life'/exp OR 'quality of life':ab,ti OR qol:ab,ti OR hrqol:ab,ti)) OR qaly*:ab,ti OR qald*:ab,ti OR qale*:ab,ti OR qtime*:ab,ti OR 'quality adjusted':ab,ti OR 'adjusted life year':ab,ti OR 'adjusted life years':ab,ti OR ('adjusted life' NEAR/3 year*):ab,ti OR 'disability adjusted life':ab,ti OR daly*:ab,ti OR ((quality OR index) NEAR/3 (wellbeing OR 'well being')):ab,ti OR qwb:ab,ti OR multiattribute*:ab,ti OR 'multi attribute':ab,ti OR (utility NEAR/2 (score* OR scoring OR valu* OR measure* OR evaluat* OR scale* OR instrument* OR weight OR weights OR weighting OR data OR unit OR units OR health OR estimate* OR elicit* OR disease* OR mean OR cost OR costs OR expenditure* OR gain OR gains OR loss OR losses OR lost OR analysis OR index* OR indices OR calculate* OR range* OR increment* OR decrement* OR decrease* OR state OR states OR status)):ab,ti OR utilities:ab,ti OR disutilit*:ab,ti OR 'dis utility':ab,ti OR 'dis utilities':ab,ti OR hsuv*:ab,ti OR ((health OR healthy) NEAR/3 (year OR years) NEAR/3 (equivalent OR equivalents)):ab,ti OR hye:ab,ti OR hyes:ab,ti OR 'health utility index':ab,ti OR 'health utilities index':ab,ti OR hui:ab,ti OR hui1:ab,ti OR hui2:ab,ti OR hui3:ab,ti OR ((illness OR health) NEAR/3 state*):ab,ti OR euroqol*:ab,ti OR euroqual*:ab,ti OR 'euro qol':ab,ti OR 'euro qual':ab,ti OR 'euro qual5d':ab,ti OR 'euro qol5d':ab,ti OR eq5d*:ab,ti OR 'eq 5d':ab,ti OR 'eq5 d':ab,ti OR 'eq 5d 5l':ab,ti OR 'eq 5d5l':ab,ti OR 'eq 5d5 l':ab,ti OR 'eq 5d 3l':ab,ti OR 'eq 5d3l':ab,ti OR 'eq 5d3 l':ab,ti OR shortform36:ab,ti OR 'short form 36':ab,ti OR shortform12:ab,ti OR 'short form 12':ab,ti OR (short NEAR/1 (form36 OR formthirtysix OR 'formthirty six' OR form6 OR form6d OR formsix OR form12 OR formtwelve)):ab,ti OR sf36*:ab,ti OR (sf NEAR/1 36*):ab,ti OR 'sf thirtysix':ab,ti OR 'sf thirty six':ab,ti OR 'sfthirtysix':ab,ti OR 'sfthirty six':ab,ti OR sf6*:ab,ti OR 'sf 6':ab,ti OR sf6d*:ab,ti OR 'sf 6d':ab,ti OR 'sf six':ab,ti OR sfsix*:ab,ti OR sf12*:ab,ti OR (sf NEAR/1 12*):ab,ti OR 'sf twelve':ab,ti OR sftwelve*:ab,ti OR 15d:ab,ti OR '15 d':ab,ti OR '15 dimension':ab,ti OR ('assessment of quality of' NEAR/1 life*):ab,ti OR 'assessment quality of life':ab,ti OR aqol*:ab,ti OR (standard NEAR/3 gamble*):ab,ti OR (sg NEAR/3 gamble):ab,ti OR timetradeoff*:ab,ti OR ('time trade' NEAR/3 off*):ab,ti OR (time NEAR/3 tradeoff*):ab,ti OR tto:ab,ti OR (visual NEAR/3 (analog OR analogue) NEAR/3 scale):ab,ti OR eqvas:ab,ti OR 'eq vas':ab,ti OR vignette*:ab,ti | 204 917 |
| #3 | #1 AND #2 | 1242 |
| #4 | disutilit*:ab,ti OR 'dis utility':ab,ti OR 'dis utilities':ab,ti OR (utility NEAR/5 (decrement* OR decrease*)):ab,ti | 1031 |
| #5 | 'lung cancer'/exp OR (lung NEAR/3 (cancer OR carcinoma)):ab,ti | 302 109 |
| #6 | #4 AND #5 | 33 |
| #7 | 'cancer growth'/exp OR 'recurrent disease'/exp OR (progress* NEAR/3 disease):ab,ti OR progression:ab,ti OR recurrence:ab,ti | 999 811 |
| #8 | 'advanced cancer'/exp OR 'inoperable cancer'/exp OR 'metastasis'/exp OR metasta*:ab,ti OR ('neoplasm'/exp OR neoplasm:ab,ti OR cancer:ab,ti OR carcinoma:ab,ti AND (advanced:ab,ti OR spread:ab,ti OR dissemination:ab,ti OR disseminated:ab,ti OR inoperable:ab,ti OR unresectable:ab,ti OR 'un resectable':ab,ti OR 'stage iv':ab,ti OR stageiv:ab,ti OR 'stage 4':ab,ti OR stage4:ab,ti OR 'stage iii':ab,ti OR stageiii:ab,ti OR 'stage 3':ab,ti OR stage3:ab,ti OR 'stage iiib':ab,ti OR stageiiib:ab,ti OR 'stage 3b':ab,ti OR stage3b:ab,ti)) | 892 294 |
| #9 | #4 AND #7 AND #8 | 45 |
| #10 | 'adverse drug reaction'/exp OR toxicity:ab,ti OR toxicities:ab,ti OR ae:ab,ti OR aes:ab,ti OR ((adverse OR side OR grade OR '3 4' OR 'iii iv') NEAR/2 (event* OR effect*)):ab,ti | 1 288 102 |
| #11 | 'cancer therapy'/exp OR 'antineoplastic agent'/exp OR chemotherapy:ab,ti OR immunotherapy:ab,ti OR (cancer NEAR/3 (therap* OR treatment*)):ab,ti | 2 214 095 |
| #12 | #4 AND #10 AND #11 | 69 |
| #13 | 'liver'/exp OR 'liver cancer'/exp OR 'nervous system'/exp OR 'central nervous system metastasis'/exp OR 'adrenal gland'/exp OR 'adrenal metastasis'/exp OR 'neoplasms of the thorax and thoracic cavity'/exp OR 'bone'/exp OR 'bone tumor'/exp OR liver:ab,ti OR hepatic:ab,ti OR nervous:ab,ti OR cns:ab,ti OR adrenal:ab,ti OR respiratory:ab,ti OR chest:ab,ti OR thorax:ab,ti OR thoracic:ab,ti OR intrathoracic:ab,ti OR extrathoracic:ab,ti OR 'extra thoracic':ab,ti OR bone:ab,ti OR bones:ab,ti | 5 574 911 |
| #14 | #4 AND #8 AND #13 | 22 |
| #15 | 'neutropenia'/exp OR 'infection'/exp OR 'cancer fatigue'/exp OR 'chemotherapy induced nausea and vomiting'/exp OR 'diarrhea'/exp OR 'gastrointestinal symptom'/exp OR 'alopecia'/exp OR 'bleeding'/exp OR 'hypertension'/exp OR 'rash'/exp OR 'ulcer'/exp OR 'stomatitis'/exp OR 'visual disorder'/exp OR 'self esteem'/exp OR 'anemia'/exp OR 'inflammation of the lungs, bronchi and pleura'/exp OR 'aspartate aminotransferase'/exp OR 'fracture'/exp OR febrile:ab,ti OR fever:ab,ti OR neutropeni*:ab,ti OR neutropaeni*:ab,ti OR infection*:ab,ti OR sepsis:ab,ti OR septicaemia:ab,ti OR septicemia:ab,ti OR fatigue:ab,ti OR lethargy:ab,ti OR lethargic:ab,ti OR nausea:ab,ti OR vomiting:ab,ti OR diarrhoea*:ab,ti OR diarrhea*:ab,ti OR gastrointestinal:ab,ti OR 'gastro intestinal':ab,ti OR (gi NEAR/1 (complaint* OR discomfort OR distress OR disturbance* OR problem* OR 'side effect' OR 'side effects' OR symptom* OR upset)):ab,ti OR 'hair loss':ab,ti OR alopecia:ab,ti OR bleeding:ab,ti OR haemorrhage:ab,ti OR hemorrhage:ab,ti OR hypertension:ab,ti OR 'high blood pressure':ab,ti OR rash:ab,ti OR ulcer*:ab,ti OR stomatitis:ab,ti OR 'sore mouth':ab,ti OR 'cancrum oris':ab,ti OR ((mouth OR oral) NEAR/3 inflammation):ab,ti OR mucositis:ab,ti OR ((visual OR vision OR sight) NEAR/3 (disorder* OR disturbance* OR abnormalit* OR subnormal OR loss OR acuity OR impaired OR impairment OR handicap*)):ab,ti OR 'self esteem':ab,ti OR 'self perception':ab,ti OR 'self concept':ab,ti OR (psycholog* NEAR/3 change*):ab,ti OR anaemia:ab,ti OR anemia:ab,ti OR pneumonia*:ab,ti OR pneumonitis:ab,ti OR ast:ab,ti OR 'aspartate aminotransferase':ab,ti OR 'aspartate transaminase':ab,ti OR fracture:ab,ti OR ('skeletal related' NEAR/3 event*):ab,ti | 6 154 666 |
| #16 | 'quality of life'/exp OR 'health status'/exp OR 'cancer patient'/exp | 652 192 |
| #17 | #8 OR #11 OR #16 | 3 166 656 |
| #18 | #4 AND #15 AND #17 | 216 |
| #19 | #6 OR #9 OR #12 OR #14 OR #18 | 300 |
| #20 | #3 OR #19 | 1521 |

**Databases: MEDLINE In-Process and e-publications ahead of print**

Date search run: 7 September 2016

Hits: 144 (from search on 7 September 2016)

Utilities filter: adapted from Arber 2015 [30]

| **No.** | **Query** | **Results** |
| --- | --- | --- |
| #1 | Search ("Carcinoma, Non-Small-Cell Lung"[mh] OR NSCLC*[tiab] OR mNSCLC*[tiab] OR aNSCLC*[tiab] OR msqNSCLC*[tiab] OR “non sq NSCLC” [tiab] OR SqCLC*[tiab] OR “ns NSCLC” [tiab] OR nsNSCLC*[tiab] OR “la NSCLC” [tiab] OR laNSCLC*[tiab] OR CPNPC*[tiab] OR (LAC[tiab] AND (lung[tiab] OR adenocarcinoma[tiab])) OR ((SCC[tiab] AND “squamous cell carcinoma” [tiab]) AND lung[tiab]) OR (non[tiab] AND small[tiab] AND cell[tiab] AND lung[tiab] AND (cancer*[tiab] OR carcinoma*[tiab])) OR ((“non small” [tiab] OR nonsmall[tiab]) AND lung[tiab] AND cell[tiab] AND (cancer*[tiab] OR carcinoma*[tiab])) OR ((“non small” [tiab] OR nonsmall[tiab]) AND cell[tiab] AND lung[tiab] AND (cancer*[tiab] OR carcinoma*[tiab])) OR (bronchial[tiab] AND (“non small” [tiab] OR nonsmall[tiab]) AND cell[tiab] AND (cancer*[tiab] OR carcinoma*[tiab])) OR (“non small cell”[tiab] AND (lung[tiab] OR bronchial[tiab] OR pulmonary[tiab] OR bronchopulmonary[tiab] OR bronchus[tiab]) AND (cancer*[tiab] OR carcinoma*[tiab])) OR (“non small” [tiab] AND cell[tiab] AND (cancer*[tiab] OR carcinoma*[tiab]) AND lung*[tiab]) OR (pulmonary[tiab] AND “non small cell” [tiab] AND (cancer*[tiab] OR carcinoma*[tiab])) OR (large[tiab] AND cell[tiab] AND lung[tiab] AND (cancer*[tiab] OR carcinoma*[tiab])) OR ((squamous[tiab] OR nonsquamous[tiab] OR “non squamous” [tiab]) AND (cell[tiab] OR “non small cell” [tiab]) AND lung[tiab] AND (cancer*[tiab] OR carcinoma*[tiab])) OR (bronchus[tiab] AND squamous[tiab] AND cell[tiab] AND (cancer*[tiab] OR carcinoma*[tiab])) OR (lung[tiab] AND epidermoid[tiab] AND (cancer*[tiab] OR carcinoma*[tiab])) OR (lung[tiab] AND squamous[tiab] AND cell[tiab] AND (cancer*[tiab] OR carcinoma*[tiab])) OR ((lung[tiab] OR poumon[tiab]) AND (NSCLC*[tiab] OR CPNPC*[tiab] OR “non small” [tiab] OR nonsmall[tiab] OR large[tiab] OR squamous[tiab] OR “non squamous” [tiab] OR nonsquamous[tiab] OR “non à petites cellules” [tiab]) AND (neoplasm*[tiab] OR cancer*[tiab] OR carcinoma*[tiab] OR adenocarcinoma*[tiab] OR angiosarcoma*[tiab] OR chrondosarcoma*[tiab] OR sarcoma*[tiab] OR teratoma*[tiab] OR lymphoma*[tiab] OR blastoma*[tiab] OR microcytic*[tiab] OR carcinogenesis[tiab] OR tumour*[tiab] OR tumor*[tiab] OR metasta*[tiab] OR métastasé[tiab] OR métastatique[tiab] OR avancé[tiab] OR “progression localisée”[tiab])) OR ((adenocancer[tiab] OR adenocarcinoma[tiab]) AND (lung[tiab] OR pulmonary[tiab])) OR (((cancer[tiab] OR tumeur[tiab]) AND (poumon[tiab] OR bronchique[tiab])) AND (“non à petites cellules”[tiab] OR “non-lié à de petites cellules”[tiab]))) | 88 016 |
| #2 | Search ("Quality-Adjusted Life Years"[mh] OR "Value of Life"[mh] OR (utility[tiab] AND (“quality of life”[mh] OR “quality of life”[tiab] OR qol[tiab] OR hrqol[tiab])) OR (qaly*[tiab] OR qald*[tiab] OR qale*[tiab] OR qtime*[tiab] OR “quality adjusted”[tiab] OR “adjusted life year”[tiab] OR “adjusted life years”[tiab] OR (“adjusted life”[tiab] AND year*[tiab]) OR “disability adjusted life” [tiab] OR daly*[tiab] OR ((quality[tiab] OR index[tiab]) AND (wellbeing[tiab] OR “well being” [tiab])) OR qwb[tiab] OR multiattribute*[tiab] OR “multi attribute”[tiab] OR (utility[tiab] AND (score*[tiab] OR scoring[tiab] OR valu*[tiab] OR measure*[tiab] OR evaluat*[tiab] OR scale*[tiab] OR instrument*[tiab] OR weight[tiab] OR weights[tiab] OR weighting[tiab] OR data[tiab] OR unit[tiab] OR units[tiab] OR health[tiab] OR estimate*[tiab] OR elicit*[tiab] OR disease*[tiab] OR mean[tiab] OR cost[tiab] OR costs[tiab] OR expenditure*[tiab] OR gain[tiab] OR gains[tiab] OR loss[tiab] OR losses[tiab] OR lost[tiab] OR analysis[tiab] OR index*[tiab] OR indices[tiab] OR calculate*[tiab] OR range*[tiab] OR increment*[tiab] OR decrement*[tiab] OR decrease*[tiab] OR state[tiab] OR states[tiab] OR status[tiab])) OR (utility[tiab] AND (“self efficacy”[tiab] OR “social support”[tiab])) OR utilities[tiab] OR disutilit*[tiab] OR “dis utility”[tiab] OR “dis utilities”[tiab] OR HSUV*[tiab] OR ((health[tiab] OR healthy[tiab]) AND (year[tiab] OR years[tiab]) AND (equivalent[tiab] OR equivalents[tiab])) OR hye[tiab] OR hyes[tiab] OR “health utility index”[tiab] OR “health utilities index”[tiab] OR hui[tiab] OR hui1[tiab] OR hui2[tiab] OR hui3[tiab] OR ((illness[tiab] OR health[tiab]) AND state*[tiab]) OR euroqol*[tiab] OR euroqual*[tiab] OR “euro qol”[tiab] OR “euro qual”[tiab] OR “euro qual5d”[tiab] OR “euro qol5d”[tiab] OR eq5d*[tiab] OR “eq 5d”[tiab] OR “eq5 d”[tiab] OR “eq 5d 5l”[tiab] OR “eq 5d5l”[tiab] OR “eq 5d5 l”[tiab] OR “eq 5d 3l”[tiab] OR “eq 5d3l”[tiab] OR “eq 5d3 l”[tiab] OR shortform36[tiab] OR “short form 36”[tiab] OR shortform12[tiab] OR “short form 12”[tiab] OR (short[tiab] AND (form36[tiab] OR formthirtysix[tiab] OR “formthirty six”[tiab] OR form6[tiab] OR form6d[tiab] OR formsix[tiab] OR form12[tiab] OR formtwelve[tiab])) OR sf36*[tiab] OR (sf[tiab] AND 36[tiab]) OR “sf thirtysix”[tiab] OR “sf thirty six”[tiab] OR “sfthirtysix”[tiab] OR “sfthirty six”[tiab] OR sf6*[tiab] OR “sf 6”[tiab] OR sf6d*[tiab] OR “sf 6d”[tiab] OR “sf six”[tiab] OR sfsix*[tiab] OR sf12*[tiab] OR (sf[tiab] AND 12[tiab]) OR “sf twelve”[tiab] OR sftwelve*[tiab] OR 15d[tiab] OR “15 d”[tiab] OR “15 dimension”[tiab] OR (“assessment of quality of”[tiab] AND life*[tiab]) OR “assessment quality of life”[tiab] OR aqol*[tiab] OR (standard[tiab] AND gamble*[tiab]) OR (sg[tiab] AND gamble[tiab]) OR timetradeoff*[tiab] OR (“time trade”[tiab] AND off[tiab]) OR (time[tiab] AND tradeoff*[tiab]) OR tto[tiab] OR (visual[tiab] AND analogue[tiab] AND scale[tiab]) OR EQVAS[tiab] OR “EQ VAS”[tiab] OR vignette*[tiab]) | 385 637 |
| #3 | Search (#1 AND #2) | 1663 |
| #4 | Search (disutility*[tiab] OR “dis utility”[tiab] OR “dis utilities”[tiab] OR (utility[tiab] AND (decrement*[tiab] OR decrease*[tiab]))) | 10 138 |
| #5 | Search ("Lung Neoplasms"[mh] OR (lung[tiab] AND (cancer[tiab] OR carcinoma[tiab]))) | 255 364 |
| #6 | Search (#4 AND #5) | 177 |
| #7 | Search (“disease progression”[mh] OR (progress*[tiab] AND disease[tiab]) OR progression[tiab] OR recurrence[tiab]) | 763 162 |
| #8 | Search ("Neoplasm Metastasis"[mh] OR metasta*[tiab] OR ((“neoplasms”[mh] OR neoplasm[tiab] OR cancer[tiab] OR carcinoma[tiab]) AND (advanced[tiab] OR spread[tiab] OR dissemination[tiab] OR disseminated[tiab] OR inoperable[tiab] OR unresectable[tiab] OR “un resectable”[tiab] OR “stage iv”[tiab] OR stageiv[tiab] OR “stage 4”[tiab] OR stage4[tiab] OR “stage iii”[tiab] OR stageiii[tiab] OR “stage 3”[tiab] OR stage3[tiab] OR “stage iiib”[tiab] OR stageiiib[tiab] OR “stage 3b”[tiab] OR stage3b[tiab]))) | 608 633 |
| #9 | Search (#4 AND #7 AND #8) | 178 |
| #10 | Search ("Drug-Related Side Effects and Adverse Reactions"[mh] OR toxicity[tiab] OR toxicities[tiab] OR AE[tiab] OR AEs[tiab] OR ((adverse[tiab] OR side[tiab] OR grade[tiab] OR “3 4”[tiab] OR “III IV”[tiab]) AND (event*[tiab] OR effect*[tiab]))) | 1 033 475 |
| #11 | Search ("Antineoplastic Protocols"[mh] OR "Antineoplastic Agents"[mh] OR chemotherapy[tiab] OR immunotherapy[tiab] OR (cancer[tiab] AND (therap*[tiab] OR treatment*[tiab]))) | 992 435 |
| #12 | Search (#4 AND #10 AND #11) | 233 |
| #13 | Search (“liver”[mh] OR “liver neoplasms”[mh] OR “nervous system”[mh] OR “central nervous system neoplasms”[mh] OR “adrenal glands”[mh] OR “thoracic neoplasms”[mh] OR “bone and bones”[mh] OR “bone neoplasms”[mh] OR liver[tiab] OR hepatic[tiab] OR nervous[tiab] OR CNS[tiab] OR adrenal[tiab] OR respiratory[tiab] OR chest[tiab] OR thorax[tiab] OR thoracic[tiab] OR intrathoracic[tiab] OR extrathoracic[tiab] OR “extra thoracic”[tiab] OR bone[tiab] OR bones[tiab]) | 4 508 746 |
| #14 | Search (#4 AND #8 AND #13) | 193 |
| #15 | Search (“neutropenia”[mh] OR “infection”[mh] OR “fatigue”[mh] OR "Fatigue Syndrome, Chronic"[mh] OR “nausea”[mh] OR “vomiting”[mh] OR “diarrhea”[mh] OR “gastrointestinal diseases”[mh] OR “alopecia”[mh] OR “hemorrhage”[mh] OR “hypertension”[mh] OR “exanthema”[mh] OR “ulcer”[mh] OR “stomatitis”[mh] OR “vision disorders”[mh] OR “self concept”[mh] OR “anemia”[mh] OR “pneumonia”[mh] OR “aspartate aminotransferases”[mh] OR “fractures, bone”[mh] OR febrile[tiab] OR fever[tiab] OR neutropeni*[tiab] OR neutropaeni*[tiab] OR infection*[tiab] OR sepsis[tiab] septicaemia[tiab] OR septicaemia[tiab] OR fatigue[tiab] OR lethargy[tiab] OR lethargic[tiab] OR nausea[tiab] OR vomiting[tiab] OR diarrhoea[tiab] OR diarrhea[tiab] OR gastrointestinal[tiab] OR “gastro intestinal”[tiab] OR (GI[tiab] AND (complaint*[tiab] OR discomfort[tiab] OR distress[tiab] OR disturbance*[tiab] OR problem*[tiab] OR “side effect” [tiab] OR “side effects”[tiab] OR symptom*[tiab] OR upset[tiab])) OR “hair loss”[tiab] OR alopecia[tiab] OR bleeding[tiab] OR haemorrhage[tiab] OR hemorrhage[tiab] OR hypertension[tiab] OR “high blood pressure”[tiab] OR rash[tiab] OR exanthema*[tiab] OR ulcer*[tiab] OR stomatitis[tiab] OR “sore mouth” [tiab] OR “cancrum oris” [tiab] OR ((mouth[tiab] OR oral[tiab]) AND inflammation[tiab]) OR mucositis[tiab] OR ((visual[tiab] OR vision[tiab] OR sight[tiab]) AND (disorder*[tiab] OR disturbance*[tiab] OR abnormalit*[tiab] OR subnormal[tiab] OR loss[tiab] OR acuity[tiab] OR impaired[tiab] OR impairment[tiab] OR handicap*[tiab])) OR “self esteem”[tiab] OR “self perception”[tiab] OR “self concept”[tiab] OR (psycholog*[tiab] AND change*[tiab]) OR anaemia[tiab] OR anemia[tiab] OR pneumonia*[tiab] OR pneumonitis[tiab] OR AST[tiab] OR “aspartate aminotransferase”[tiab] OR “aspartate transaminase”[tiab] OR fracture[tiab] OR (“skeletal related”[tiab] AND event*[tiab])) | 1 728 321 |
| #16 | Search (“quality of life”[mh] OR “health status”[mh]) | 250 313 |
| #17 | Search (#4 AND (#8 OR #11 OR #16) AND #15) | 220 |
| #18 | Search (#3 OR #6 OR #9 OR #12 OR #14 OR #17) | 2317 |
| #19 | Search (pubstatusaheadofprint[tiab] OR inprocess[sb]) | 676 718 |
| #20 | Search (#18 AND #19) | 144 |

**Databases: Cochrane Library**

**Cochrane Database of Systematic Reviews (CDSR): Issue 9 of 12, September 2016**

**Database of Abstracts of Reviews of Effects (DARE): Issue 2 of 4, April 2015**

**Cochrane Central Register of Controlled Trials (CENTRAL): Issue 8 of 12, August 2016**

**NHS Economic Evaluation Database (NHS EED): Issue 2 of 4, April 2015**

**Health Technology Assessment Database (HTAD): Issue 3 of 4, July 2016**

Date search run: 7 September 2016

Hits: CDSR 0, DARE 0, CENTRAL 174, HTAD 1, NHS EED 43

Utilities filter adapted from Arber*.* 2015 [30]

| **No.** | **Query** | **Results** |
| --- | --- | --- |
| #1 | MeSH descriptor: [Carcinoma, Non-Small-Cell Lung] explode all trees | 2824 |
| #2 | (NSCLC* or mNSCLC* or aNSCLC* or msqNSCLC* or "non sq NSCLC" or SqCLC* or "ns NSCLC" or nsNSCLC* or "la NSCLC" or laNSCLC* or CPNPC* or (LAC near/3 (lung or adenocarcinoma)) or ((SCC near/3 "squamous cell carcinoma") and lung) or (non near/3 small near/3 cell near/3 lung near/3 (cancer* or carcinoma*)) or (("non small" or nonsmall) near/3 lung near/3 cell near/3 (cancer* or carcinoma*)) or (("non small" or nonsmall) near/3 cell near/3 lung near/3 (cancer* or carcinoma*)) or (bronchial near/3 ("non small" or nonsmall) near/3 cell near/3 (cancer* or carcinoma*)) or ("non small cell" near/3 (lung or bronchial or pulmonary or bronchopulmonary or bronchus) near/3 (cancer* or carcinoma*)) or ("non small" near/3 cell near/3 (cancer* or carcinoma*) near/3 lung*) or (pulmonary near/3 "non small cell" near/3 (cancer* or carcinoma*)) or (large near/3 cell near/3 lung near/3 (cancer* or carcinoma*)) or ((squamous or nonsquamous or "non squamous") near/5 (cell or "non small cell") near/3 lung near/3 (cancer* or carcinoma*)) or (bronchus near/3 squamous near/3 cell near/3 (cancer* or carcinoma*)) or (lung near/3 epidermoid near/3 (cancer* or carcinoma*)) or (lung near/3 squamous near/3 cell near/3 (cancer* or carcinoma*)) or ((lung or poumon) and (NSCLC* or CPNPC* or "non small" or nonsmall or large or squamous or "non squamous" or nonsquamous or "non à petites cellules") and (neoplasm* or cancer* or carcinoma* or adenocarcinoma* or angiosarcoma* or chrondosarcoma* or sarcoma* or teratoma* or lymphoma* or blastoma* or microcytic* or carcinogenesis or tumour* or tumor* or metasta* or métastasé or métastatique or avancé or "progression localisée")) or ((adenocancer or adenocarcinoma) near/3 (lung or pulmonary)) or (((cancer or tumeur) near/3 (poumon or bronchique)) and ("non à petites cellules" or "non-lié à de petites cellules"))):ti,ab,kw | 7164 |
| #3 | #1 or #2 | 7164 |
| #4 | #3 in Technology Assessments and Economic Evaluations | 312 |
