# Supplementary material for: Systematic review of health state utility values in metastatic non-small cell lung cancer with a focus on previously treated patients
Source: Health Qual Life Outcomes. 2018 Sep 12;16:179. doi: 10.1186/s12955-018-0994-8 (PMC6134713; doi:10.1186/s12955-018-0994-8)
Supplement: Supplementary file 2 — Table S2. Listing of first-line mNSCLC studies with utility data excluded at second pass [80–100]. (DOCX 84 kb) [file 12955_2018_994_MOESM2_ESM.docx]

# Additional File 2 – Table S2 Listing of first-line (treatment-naïve and maintenance first-line) mNSCLC studies with utility data excluded at second pass

These studies were included for reference at first pass screening but did not undergo data extraction.

| **Author, year, reference** | **Title** | **Citation** |
| --- | --- | --- |
| Balçik 2016 [80] | Cost-effectiveness analysis of pemetrexed and gemcitabine treatment for advanced nonsmall cell lung cancer in turkey | Turk J Med Sci. 2016;46:152–158 |
| Barney 2013 [81] | Prognostic value of patient-reported symptom interference in patients with late-stage lung cancer | Qual Life Res. 2013;22:2143–50 |
| Belani 2006 [82] | Effect of chemotherapy for advanced non-small cell lung cancer on patients' quality of life. A randomized controlled trial | Lung Cancer. 2006;53:231–39 |
| Billingham 2011 [83] | Quality of life in advanced non-small cell lung cancer, effects of cisplatin dose and carboplatin in combination with gemcitabine: Results from BTOG2, a British thoracic oncology group phase III trial in 1363 patients | J Thorac Oncol. 2011;6:S319–20 |
| Chouaid 2011a [84] | Real life outcomes and health-related quality of life (HRQOL) in 1st line non-squamous non-small cell lung cancer (NSCLC): a European pilot study analysing bevacizumab-based versus non-bevacizumab-based treatments | J Thorac Oncol. 2011;6:S1284–5 |
| Chouaid 2011b [85] | Health-related quality of life (HRQOL) in 1st line non-squamous non-small cell lung cancer (NSCLC) patients in a real life setting: bevacizumab-based versus non-bevacizumab based therapy in a European pilot study | Value Health. 2011;14:A171 |
| Dranitsaris 2002 [86] | Cost-effectiveness of chemotherapy for non-small cell lung cancer | Curr Opin Oncol. 2002;14:375–83 |
| Felip 2015 [87] | Impact of crizotinib on patient-reported general health status compared with chemotherapy in patients with no prior systemic treatment for advanced non-squamous ALK-positive non-small cell lung cancer (NSCLC) | J Clin Oncol. 2015:33:8101 |
| Fleeman 2010 [88] | Pemetrexed for the first-line treatment of locally advanced or metastatic non-small cell lung cancer | Health Technol Assessment. 2010;14:47–53 |
| Galetta 2015 [89] | Cisplatin/pemetrexed followed by maintenance pemetrexed versus carboplatin/paclitaxel/bevacizumab followed by maintenance bevacizumab in advanced non-squamous lung cancer: the GOIM (Gruppo Oncologico Italia Meridionale) ERACLE phase III randomized trial | Clin Lung Cancer. 2015;16:262–73 |
| Galetta 2013 [90] | First-line pemetrexed plus cisplatin followed by maintenance pemetrexed versus carboplatin-paclitaxel plus bevacizumab followed by maintenance bevacizumab (ERACLE) in advanced non-squamous NSCLC: a quality-of-life-oriented, multicenter randomized phase III trial of the GOIM (Gruppo Oncologico Italia Meridionale) | J Thorac Oncol. 2013;8:S1002 |
| Gridelli 2012 [91] | Safety, resource use, and quality of life in paramount: A phase III study of maintenance pemetrexed versus placebo after induction pemetrexed plus cisplatin for advanced non-squamous non-small-cell lung cancer | J Thorac Oncol. 2012;7:1713–21 |
| Hirsh 2016 [92] | First-line afatinib (A) vs gefitinib (G) for patients (pts) with EGFR mutation positive (EGFRm+) NSCLC (LUX-Lung 7): patient-reported outcomes (PROs) and impact of dose modifications on efficacy and adverse events (AEs) | J Clin Oncol. 2016:34:S9046 |
| Khan 2015 [93] | Cost-effectiveness of first-line erlotinib in patients with advanced non-small-cell lung cancer unsuitable for chemotherapy | BMJ Open. 2015;5:e006733 |
| Lal 2015 [94] | Feasibility of home delivery of pemetrexed in patients with advanced non-squamous non-small cell lung cancer | Lung Cancer. 2015;89:154–60 |
| Langer 2016 [95] | Quality of life (QoL) by response: An interim analysis of patients (pts) with squamous (SCC) NSCLC treated with nab-paclitaxel/carboplatin (nab-P/C) induction therapy in the phase III ABOUND.Sqm study | J Clin Oncol. 2016;34:S64 |
| Langer 2015 [96] | Survival, quality-adjusted survival, and other clinical end points in older advanced non-small-cell lung cancer patients treated with albumin-bound paclitaxel | Br J Cancer. 2015;113:20–9 |
| Reck 2016 [97] | The effect of necitumumab in combination with gemcitabine plus cisplatin on tolerability and on quality of life: results from the phase 3 SQUIRE trial | J Thorac Oncol. 2016;11:808–18 |
| Shallwani 2016 [98] | Quality of life, symptom status and physical performance in patients with advanced non-small cell lung cancer undergoing chemotherapy: an exploratory analysis of secondary data | Lung Cancer. 2016;99:69–75 |
| Tongpak 2012 [99] | Utility of advanced non-small cell lung cancer patients in Thailand: preliminary study | Value Health. 2012;15:A657 |
| Yalçin Balçik 2016 [100] | Cost-effectiveness analysis of pemetrexed and gemcitabine treatment for advanced non-small cell lung cancer in Turkey | Turk J Med Sci. 2016;46:152–158 |
