# Supplementary material for: Systematic review of health state utility values in metastatic non-small cell lung cancer with a focus on previously treated patients
Source: Health Qual Life Outcomes. 2018 Sep 12;16:179. doi: 10.1186/s12955-018-0994-8 (PMC6134713; doi:10.1186/s12955-018-0994-8)
Supplement: Supplementary file 3 — Figure S1. Hierarchy of preferred methodology for generation of HSUVs for different HTA agencies. (PDF 1172 kb) [file 12955_2018_994_MOESM3_ESM.pdf]

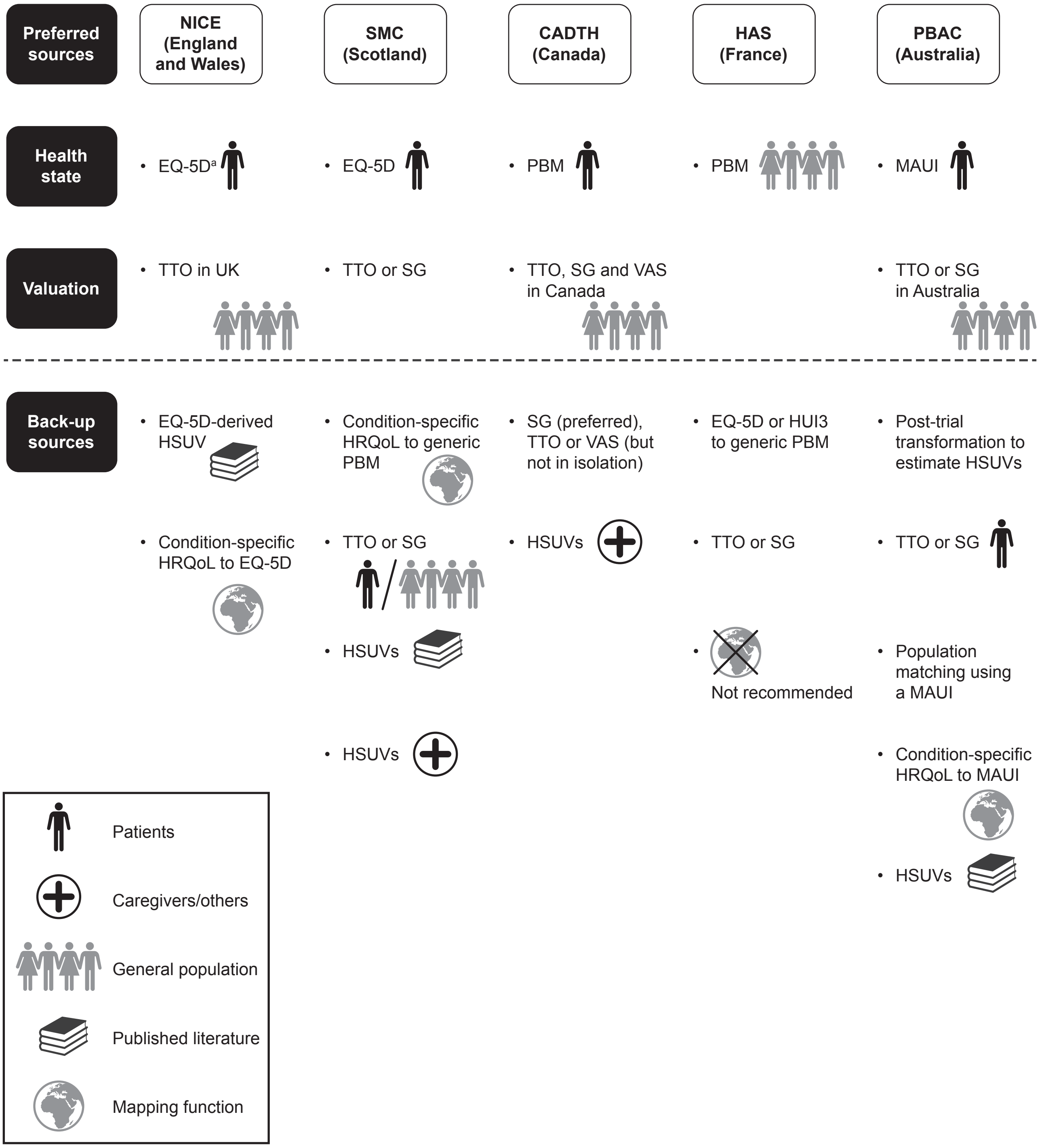

Preferred sources
NICE (England and Wales)
SMC (Scotland)
CADTH (Canada)
HAS (France)
PBAC (Australia)
Health state
EQ-5D[a]
EQ-5D
PBM
PBM
MAUI
Valuation
TTO in UK
TTO or SG
TTO, SG and VAS in Canada
TTO or SG in Australia
Back-up sources
EQ-5D-derived HSUV
Condition-specific HRQoL to EQ-5D
Condition-specific HRQoL to generic PBM
TTO or SG
HSUVs
HSUVs
SG (preferred), TTO or VAS (but not in isolation)
HSUVs
EQ-5D or HUI3 to generic PBM
TTO or SG
Not recommended
Post-trial transformation to estimate HSUVs
TTO or SG
Population matching using a MAUI
Condition-specific HRQoL to MAUI
HSUVs
Patients
Caregivers/others
General population
Published literature
Mapping function
