# Supplementary material for: Systematic review of health state utility values in metastatic non-small cell lung cancer with a focus on previously treated patients
Source: Health Qual Life Outcomes. 2018 Sep 12;16:179. doi: 10.1186/s12955-018-0994-8 (PMC6134713; doi:10.1186/s12955-018-0994-8)
Supplement: Supplementary file 4 — Table S3. Quality assessment of identified studies. (DOCX 111 kb) [file 12955_2018_994_MOESM4_ESM.docx]

# Additional File 4 – Table S3 Quality assessment of identified studies

| **Study** | **Comments on quality of study** | | | |
| --- | --- | --- | --- | --- |
|  | **Sample size, *n*** | **Measure of variability** | **Response rate, %** | **Other quality indicators, e.g. loss to follow-up, handling of missing data** |
| ***First line*** | | | | |
| Handorf 2012 [[70](#_ENREF_70)] | NR | NR | NR | Lowest in hierarchy of estimates based on expert opinion |
| Nafees 2016 [[68](#_ENREF_68)] | 451 | NR | NR | NR |
| ***≥ First line*** | | | | |
| Chevalier 2013 [[38](#_ENREF_38)] | 258 | SD | 80 | NR |
| Chouaid 2013 [[39](#_ENREF_39)] | 263 | SD and CI | 82 | NR |
| Iyer 2013 [[46](#_ENREF_46)] | 1213 | SD | 69 | NR |
| ***Second line*** | | | | |
| Blackhall 2014 [[41](#_ENREF_41)] | 172 (CRZ), 99 (PEM), 72 (DOC) | SD, SE and CI | 92–97 | Patients who had completed ≥ 1 question at BL and ≥ 1 time point on treatment were included |
| Huang 2016 [[45](#_ENREF_45)] | 560 | CI | NR | NR |
| Langley 2013 [[48](#_ENREF_48)] | 151 | NR | ≥ 80 | NR |
| Lloyd 2008 [[59](#_ENREF_59)] | 85 (SG), 26 (TTO) | CI | 95 (SG), 53 (TTO) | NR |
| Nafees 2008 [[69](#_ENREF_69)] | 100 | SE | 95 | NR |
| Novello 2015 [[49](#_ENREF_49)] | 1314 | NR | > 80 | NR |
| Reck 2015 [[50](#_ENREF_50)] | NR | SD | 64–72 | NR |
| Rudell 2016 [[57](#_ENREF_57)] | 175 (BL), 102 (week 36) | SD | NR | NR |
| Schuette 2012 [[51](#_ENREF_51)] | 521 | SD | ~ 50 | NR |
| Vargas 2009 [[72](#_ENREF_72)] | 24 (ERL), 24 (taxanes) | NR | NR | NR |
| Westwood 2014 [[71](#_ENREF_71)] | NR | SE | NR | NR |
| ***≥ Second line*** | | | | |
| Chen 2010 [[64](#_ENREF_64)] | NR | NR | N/A | N/A |
| Griebsch 2014 [[37](#_ENREF_37)] | 585 (LUX-LING 1),  345 (LUX-LING 3) | NR | 65–100 (LUX-LING 1),  > 90 (LUX-LING 3) | Attrition greater in control arms (LUX‑LING 1)  Missing data due to attrition not considered which may introduce bias (LUX-LING 3) |
| Hirsh 2013 [[40](#_ENREF_40)] | 585 | NR | 65–100 (up to week 24/end of treatment)  45–46 (during first follow-up visit) | If a patient-reported outcome assessment was missed but followed by another assessment and deterioration occurred during that time period, the time to deterioration was defined as the midpoint between the two observed assessments. Modelling undertaken to evaluate uncertainty associated with missing data |
| Schwartzberg 2015 [[60](#_ENREF_60)] | 557 (week 6), 46 (week 30) | SD | NR | NR |
| Stewart 2015 [[56](#_ENREF_56)] | 55 | SE | NR | NR |
| ***Treatment line not specified*** | | | | |
| Bradbury 2008 [[42](#_ENREF_42)] | 31 (ERL), 33 (BSC) | NR | NR | NR |
| Chang 2016 [[63](#_ENREF_63)] | 205 | CI | NR | NR |
| Dansk 2016 [[43](#_ENREF_43)] | N/A | Range | NR | NR |
| Doyle 2008 [[65](#_ENREF_65)] | 101 | SE | NR | NR |
| Grunberg 2009 [[58](#_ENREF_58)] | NR | NR | NR | NR |
| Grutters 2010 [[44](#_ENREF_44)] | 260 | NR | 70 | NR |
| Jang 2010 [[47](#_ENREF_47)] | 172 | NR | NR | NR |
| Linnet 2015 [[62](#_ENREF_62)] | 45 | NR | NR | NR |
| Lloyd 2005 [[66](#_ENREF_66)] | 78 | NR | NR | NR |
| Manser 2006 [[61](#_ENREF_61)] | 40 | IQR | 96–98 (operable)  73–76 (inoperable) | NR |
| Matza 2014 [[67](#_ENREF_67)] | 187 | SD | NR | NR |
| Tabberer 2006 [[52](#_ENREF_52)] | 154 | NR | NR | NR |
| Trippoli 2001 [[53](#_ENREF_53)] | 95 | SD | 100 | NR |
| Yang 2014 [[54](#_ENREF_54)] | 518 | SD | NR | Individuals with incomplete data (n = 20) or no information of performance status (n = 108) were not included |
| Yokoyama 2013 [[55](#_ENREF_55)] | 274 (total), 9 (utility) | NR | NR | NR |

*Abbreviations: BL* baseline, *BSC* best supportive care, *CI* confidence interval, *CRZ* crizotinib, *DOC* docetaxel, *ERL* erlotinib, *IQR* interquartile range, *N/A* not applicable, *NR* not reported, *PEM* pemetrexed, *SD* standard deviation, *SE* standard error, *SG* standard gamble, *TTO* time trade-off
